# ﻿Taxonomy and phylogeny of the genus *Ganoderma* (Polyporales, Basidiomycota) in Costa Rica

**DOI:** 10.3897/mycokeys.100.106810

**Published:** 2023-11-13

**Authors:** Melissa Mardones, Julieta Carranza-Velázquez, Milagro Mata-Hidalgo, Xaviera Amador-Fernández, Hector Urbina

**Affiliations:** 1 Escuela de Biología, Universidad de Costa Rica, San Pedro de Montes de Oca, 11501-2060, San José, Costa Rica; 2 Herbario Luis Fournier Origgi (USJ), Centro de Investigación en Biodiversidad y Ecología Tropical (CIBET), Universidad de Costa Rica, San Pedro de Montes de Oca, 11501-2060, San José, Costa Rica; 3 Florida Department of Agriculture and Consumer Services, Division of Plant Industry, Section of Plant Pathology, 1911 SW 34th St, Gainesville, Florida, 32608, USA

**Keywords:** Central America, fungal diversity, ITS, key neotropical species

## Abstract

*Ganoderma* species are well recognised by their significant role in the recycling of nutrients in ecosystems and by their production of secondary metabolites of medical and biotechnological importance. *Ganoderma* spp. are characterised by laccate and non-laccate, woody basidiocarps, polypore hymenophores and double-walled basidiospores generally with truncate apex. Despite the importance of this genus, its taxonomy is unclear and it includes several species’ complexes with few circumscribed species and incorrect geographic distributions. The aim of this work was to provide detailed morphological descriptions together with phylogenetic analyses using ITS sequences to confirm the presence of seven species of *Ganoderma* in Costa Rica: *G.amazonense*, *G.applanatum* s.l., *G.australe*, *G.curtisii*, *G.ecuadorense*, *G.oerstedii* and *G.parvulum*. This is the first study that integrates morphological and phylogenetic data of *Ganoderma* from Central America and a key of the neotropical species. Besides, the distribution range of *G.curtisii*, previously reported from North America and *G.ecuadorense* from South America, is expanded to Central America.

## ﻿Introduction

The genus *Ganoderma* P. Karst. (Ganodermataceae, Agaricomycetes) was erected by Karsten (1881), based on *Polyporuslucidus* (Curtis) Fr., to include species with a laccate and stipitate basidiocarp. The *Ganoderma* species are characterised by laccate and non-laccate, coriaceous to wood polypore basidiomes and double-walled basidiospores generally with a truncate apex and column-like endosporic projections (Moncalvo and Ryvarden 1997; Costa-Rezende et al. 2017). *Ganoderma* is a widely distributed genus, mostly represented by tropical species and some temperate ones; approximately 278 species have been described (Sun et al. 2022), most of them laccate (Cabarroi-Hernández et al. 2019; Index Fungorum http://www.indexfungorum.org/names/names.asp). The genus includes both ecologically and economically important species (wood decomposers, pathogens and metabolites producers of medical importance).

Due to the high phenotypic plasticity present in the *Ganoderma* species, the taxonomy of this genus is ambiguous and confusing. Several species complexes have led to few circumscribed species and incorrect geographic distributions (Moncalvo and Ryvarden 1997; Ryvarden 2000; Loyd et al. 2018; Fryssouli et al. 2020; Sun et al. 2022). Traditionally, the species delimitation within *Ganoderma* is based on basidiomata morphology and host preference. However, phylogenetic analyses using ribosomal RNA (rRNA) of global collections showed that morphological features and cultural characteristics appeared highly polyphyletic and most of the species are geographically restricted (Gottlieb et al. 2000; Loyd et al. 2018).

In the past few decades, molecular analyses have brought some clarifications for species delimitation in *Ganoderma*. Currently, only 50% of accepted *Ganoderma* species have molecular data (Sun et al. 2022). However, several studies have shown that numerous available sequences in public repositories are incorrectly annotated (Moncalvo et al. 1995; Hong and Jung 2004; Fryssouli et al. 2020). Moncalvo’s et al. paper (1995), one of the first molecular studies on *Ganoderma* using ITS sequence data, showed the delimitation of six clades in this genus, but vouchers labelled as *G.lucidum* (Curtis) P. Karst. were found scattered in five of the six clades. Fryssouli et al. (2020) found that only 40% of the ITS sequences deposited in GenBank (www.ncbi.nlm.nih.gov/Genbank) were correctly annotated.

In the Neotropics, approximately 39 species of *Ganoderma* have been reported in literature. Most of these studies were based on morphology and host associations (Ryvarden 2000, 2004; Costa-Rezende et al. 2020) and were focused on the species of a country or region, i.e. Brazil (Gomes-Silva et al. 2011; Torres-Torres et al. 2012; de Lima et al. 2014), Colombia (Bolaños et al. 2016), Ecuador (Crous et al. 2016, 2017, 2018), French West Indies (Welti and Courtecuisse 2010) and Mexico (Torres-Torres and Guzmán-Dávalos 2005; Torres-Torres et al. 2015; Cabarroi-Hernández et al. 2019). However, the circumscription of several species and their geographic distribution ranges remains largely unknown.

Recently, several studies have included molecular characterisation on some neotropical species of *Ganoderma*. Loyd et al. (2018) studied the laccate species of *Ganoderma* in the USA, using morphology, host preference data and a multilocus phylogeny employing the Internal Transcribed Spacer of the rRNA gene (ITS), elongation factor (*TEF*) and RNA polymerase II subunit 1 (*rpb1*) and subunit 2 (rpb2) and delimited four species with neotropical distribution [*G.curtisii* (Berk.) Murrill, *G.martinicense* Welti & Courtec., *G.tuberculosum* Murrill, G.cf.weberianum]. De Lima et al. (2014) and Bolaños et al. (2016), using ITS and the large subunit (LSU) of the rRNA gene, phylogenetically delimited *G.chalceum* (Cooke) Steyaert, *G.multiplicatum* (Mont.) Pat., *G.orbiforme* (Fr.) Ryvarden and *G.parvulum* Murrill from Brazil and Colombia. Cabarroi-Hernández et al. (2019), using morphology and multilocus phylogeny using ITS, *rpb2* and *TEF*, found at least two phylogenetic species (*G.mexicanum* Pat. and *G.parvulum*) within the neotropical species in the *G.weberianum*-*resinaceum* complex. Fryssouli et al. (2020) identified and curated the ITS sequences of *Ganoderma* in GenBank, including 14 neotropical species [*G.australe* (Fr.) Pat., *G.chocoense* J.A. Flores, C.W. Barnes & Ordoñez, *G.concinnum* Ryvarden, *G.curtisii*, *G.martinicense*, *G.mexicanum*, *G.multiplicatum*, *G.orbiforme*, *G.parvulum*, *G.podocarpense* J.A. Flores, C.W. Barnes & Ordoñez, *G.subfornicatum* Murrill, *G.tuberculosum* and two undescribed species of non-laccate *Ganoderma*].

There are two studies on *Ganoderma* in Costa Rica (Ruiz-Boyer 1998; Carranza & Ruiz-Boyer 2005); however, none includes molecular data or phylogenetic analyses. Nowadays, nine species have been reported in Costa Rica: *G.amazonense* Weir, *G.applanatum*, *G.australe*, *G.colossus* (Fr.) C.F. Baker, *G.lucidumsensu lato* (s.l.), *G.oerstedii* (Fr.) Torrend, *G.orbiforme*, *G.parvulum*, *G.stipitatum* (Murrill) Murrill and *G.tuberculosum* (Ruiz-Boyer 1998; Ryvarden 2004; Carranza and Ruiz-Boyer 2005; Cabarroi-Hernández et al. 2019). However, only *G.parvulum* and *G.tuberculosum* have been confirmed by molecular sequence data (Cabarroi-Hernández et al. 2019; Sun et al. 2022), while *G.stipitatum* was recently synonymised with *G.parvulum* (Cabarroi-Hernández et al. 2019). Besides, current data indicate that *G.lucidum* is restricted to Europe and only to some parts of China (Cao et al. 2012; Wang et al. 2012); hence, there is a need for confirming the diversity of *Ganoderma* of Costa Rica using both morphological and molecular analyses.

The geographical location of Costa Rica in the Central American isthmus has allowed the flow of species from North and South America, turning this country into a unique biogeographic region. Therefore, it is expected that *Ganoderma* species can be shared throughout the regions. Nevertheless, the geographic distribution of several neotropical species of *Ganoderma* is uncertain and molecular data of *Ganoderma* species from Central America is almost non-existent. The aims of this work are: I) to re-examine the species of G*anoderma* present in Costa Rica using morphology and ITS sequences of fresh collections, herbarium specimens and pure cultures; II) to describe, illustrate and expand the knowledge on distribution and biogeography of neotropical *Ganoderma* species and III) to propose a key of the neotropical species of *Ganoderma*. This study represents the first attempt to include *Ganoderma* species from Central America under morphological and phylogenetic frameworks worldwide.

## ﻿Methods

### ﻿Fungal material and morphological studies

Selected voucher collections from the Herbarium of the University of Costa Rica (**USJ**), the National Herbarium of Costa Rica (**CR**) and the Plant Industry Herbarium Gainesville (**PIHG**) of the Florida Department of Agriculture and Consumer Services (**FDACS**) were used for this study. Additionally, several specimens were collected during field trips throughout Costa Rica. In total, 370 specimens were macroscopically examined. Afterwards, 117 specimens were selected to be examined in detail, including microscopical characteristics. Representative basidiomata collected from this study have been deposited into the USJ collection. Collection sites with ecological details are mentioned together with the records below. In addition, type specimens from the United States National Herbarium (**BPI**) and The New York Botanical Garden Herbarium (**NY**) were re-examined. Overall, more than 120 specimens of nine morphotypes, including nine types, were examined.

Specimens were photographed in situ. Descriptions of macromorphological features (colour and texture of the basidiocarp and tissue context, presence/absence of stipe, melanoid deposits or concentric zones) were observed from fresh material. Microscopical preparations of the hyphal system, cuticular cells, basidiospores and chlamydospores were made in 3% potassium hydroxide (KOH), cotton blue (1 mg/ml), and Melzer’s reagent (to test dextrinoid and/or amyloid reactions). Slides were examined with a Nikon Eclipse 80i microscope with bright field and phase contrast optics. Imaging and measurements were done using a camera Nikon DS-Fi2 adapted to the microscope and operated by the Imaging Software NIS-Elements D 2.2. At least 30 individual basidiospores and chlamydospores were measured for at least three representative collections for each species. Outlying measurements observed in less than 5% of the measurements of a given structure are placed in parentheses. The number is indicated in brackets if less than 30 values were measured.

### ﻿DNA extraction, PCR and sequencing

We extracted DNA from 19 fresh specimens. Basidiome samples were ground by a Fastprep24 machine (MP Biomedicals, CA, USA). The isolation of total genomic DNA was performed using the FastDNA SPIN Kit (MP Biomedicals), following the protocol provided by the manufacturer. DNA was quantified using a Nanodrop ND-1000 spectrometer (Nanodrop Technologies, DE, USA), after which it was adjusted to a final concentration of 50 ng μl^-1^ before PCR. DNA extracts were stored in aliquots at -20 °C.

The complete ITS (ITS1-5.8S-ITS2) region with primers ITS5 and ITS4 (White et al. 1990) was amplified and sequenced. Each PCR tube contained 1 μl of DNA template, 1 μl of each primer (10 μM), 25 μl of iProof HF 2× Master Mix (BioRad, Hercules, CA, USA) and 22 μl of sterile distilled water. PCR reactions were performed on a *PEQ*STAR 2× GRADIENT Thermal Cycler (PEQLAB, Erlangen, Germany). Conditions of the PCR were as follows: DNA denaturation 98 °C for 3 min; 35 cycles of DNA denaturation 98 °C for 10 s, primer annealing 55 °C for 30 s and *Taq* extension 72 °C for 30 s and a final *Taq* extension 72 °C for 10 min, followed by storage at 8 °C. PCR-products were checked on 1.5% agarose electrophoresis gels stained with ethidium bromide. Amplified PCR products were purified with Cycle Pure Kit (VWR-Omega, GA, USA). The Sanger sequencing in both directions was performed with the same PCR primers in MACROGEN (South Korea) for the specimens collected in Costa Rica, while samples collected in Florida were sequenced in-house at the headquarters of FDACS-Division of Plant Industry in Gainesville. Additionally, one fragment of the LSU with primers NL1 and NL2 (O’Donnell 1993) and one fragment of *TEF* with primers EF1-983f and EF1-2218r (Rehner and Buckley 2005) were also amplified and sequenced, but not used in the phylogenetic analyses. The LSU and *TEF* sequences generated from Costa Rican specimens are provided and the accession numbers of these sequences are mentioned together with the records of each specimen below.

### ﻿Phylogenetic analyses

We assembled an ITS dataset comprising sequences from 159 specimens worldwide, 82 originating from the Neotropics and 15 from type specimens. This analysis aimed to infer the position of the *Ganoderma* specimens from Costa Rica in a global context. Sequences were downloaded from GenBank, mostly from studies published by Costa-Rezende et al. (2017), Loyd et al. (2018), Cabarroi-Hernández et al. (2019) and Sun et al. (2022). *Tomophaguscolossus* (Fr.) Murrill vouchers URM80450 and TC-02 were selected as outgroup taxa, based on Loyd et al. (2018). The newly-generated ITS sequences and the sequences retrieved from GenBank are given in Table 1, together with their voucher/strain numbers, location and accession numbers.

**Table 1.** Specimen data and accession numbers of the taxa used in the phylogenetic analyses. The (T) indicated type material.

Species	Voucher	ITS	Country	Reference
*Ganodermaadspersum*	GAD3	JN222418	Poland	Retrieve from GenBank
*Ganodermaadspersum*	GATO00	AM906057	Italy	Costa-Rezende et al. (2017)
*Ganodermaamazonense*	GA-54	OQ845454	Costa Rica	This study
*Ganodermaapplanatum*	Cui 14062	MZ354913	China	Sun et al. (2022)
*Ganodermaapplanatum*	Cui 14070	MZ354914	China	Sun et al. (2022)
*Ganodermaapplanatum*	GA-64	OQ845455	Costa Rica	This study
*Ganodermaapplanatum*	KM120830	AY884178	UK	Retrieve from GenBank
*Ganodermaapplanatum*	Wei5787a	KF495001	China	Retrieve from GenBank
*Ganodermaapplanatum*	SFC20141001-24	KY364255	Korea	Jargalmaa et al. (2017)
*Ganodermaapplanatum*	SFC20150930-02	KY364258	Korea	Jargalmaa et al. (2017)
*Ganodermaaridicola*	DAI 12588 (T)	KU572491	South Africa	Xing et al. (2016)
*Ganodermaaustrale*	DHCR411 (HUEFS)	MF436675	Australia	Costa-Rezende et al. (2017)
*Ganodermaaustrale*	DHCR417 (HUEFS)	MF436676	Australia	Costa-Rezende et al. (2017)
*Ganodermaaustrale*	GA-19	OQ845456	Costa Rica	This study
*Ganodermaaustroafricanum*	CBS 1387.24	KM507324	South Africa	Coetzee et al. (2015)
*Ganodermaboninense*	WD2028 (FFPRI)	KJ143905	Japan	Zhou et al. 2015
*Ganodermaboninense*	WD2085 (FFPRI)	KJ143906	Japan	Zhou et al. 2015
Ganodermacf.chocoense	GA-03	OQ845457	Costa Rica	This study
*Ganodermachocoense*	QCAM3123 (T)	MH890527	Ecuador	Crous et al. (2018)
*Ganodermaconcinnum*	Robledo 3192	MN077522	Brazil	Costa-Rezende et al. (2020)
*Ganodermaconcinnum*	Robledo 3235	MN077523	Brazil	Costa-Rezende et al. (2020)
*Ganodermacupreum*	GANOTK4	JN105701	Camerun	Retrieve from GenBank
*Ganodermacupreum*	GANOTK7	JN105702	Camerun	Retrieve from GenBank
*Ganodermacurtisii*	102NC	MG654074	NC, USA	Loyd et al. (2018)
*Ganodermacurtisii*	223FL	MG654167	FL, USA	Loyd et al. (2018)
*Ganodermacurtisii*	CBS 100132	JQ781849	NC, USA	Cao et al. (2012)
*Ganodermacurtisii*	CBS100131	JQ781848	NC, USA	Cao et al. (2012)
*Ganodermacurtisii*	GA-00	OQ845458	Costa Rica	This study
*Ganodermacurtisii*	GA-22	OQ845459	Costa Rica	This study
*Ganodermacurtisii*	GA-63	OQ845460	Costa Rica	This study
*Ganodermacurtisii*	GA-65	OQ845461	Costa Rica	This study
*Ganodermacurtisii*	P559-03202022-2284	OQ845462	FL, USA	This study
*Ganodermacurtisii*	UMNFL28	MG654097	Fl, USA	Loyd et al. (2018)
Ganodermacurtisiif.sp.meredithiae	124FL	MG654188	Fl, USA	Loyd et al. (2018)
*Ganodermaecuadorense*	Dai 17397	MZ354950	Brazil	Sun et al. (2022)
*Ganodermaecuadorense*	Dai 17418	MZ354951	Brazil	Sun et al. (2022)
*Ganodermaecuadorense*	GA-52	OQ845463	Costa Rica	This study
*Ganodermaecuadorense*	GA-57	OQ845464	Costa Rica	This study
*Ganodermaecuadorense*	JV 1808/85	MZ354952	French Guiana	Sun et al. (2022)
*Ganodermaecuadorense*	MMG-181A	OQ845465	Costa Rica	This study
*Ganodermaecuadorense*	MMG-209	OQ845466	Costa Rica	This study
*Ganodermaecuadorense*	PMC-126	KU128525	Ecuador	Crous et al. (2016)
*Ganodermaecuadorense*	Poly-2.4	KU128526	Ecuador	Crous et al. (2016)
*Ganodermaecuadorense*	QCAM3430/ASL799 (T)	KU128524	Ecuador	Crous et al. (2016)
*Ganodermaellipsoideum*	GACP14080966 (T)	MH106867	China	Hapuarachchi et al. (2018)
*Ganodermaellipsoideum*	GACP14080968	MH106868	China	Hapuarachchi et al. (2018)
*Ganodermaenigmaticum*	DAI 15970	KU572486	South Africa	Xing and Cui (2016)
*Ganodermaenigmaticum*	DAI 15971	KU572487	South Africa	Xing and Cui (2016)
*Ganodermaenigmaticum*	CBS 139792 (T)	NR_132918	South Africa	Coetzee et al. (2015)
*Ganodermaflexipes*	Wei5200	JN383978	China	Cao and Yuan (2013)
*Ganodermaflexipes*	Wei5491	JQ781850	China	Cao and Yuan (2013)
*Ganodermaflexipes*	Wei5494	JN383979	China	Cao and Yuan (2013)
*Ganodermagibbosum*	JFL14070442	MH106880	China	Hapuarachchi et al. (2018)
*Ganodermagibbosum*	KUT0805	AB733121	Japan	Costa-Rezende et al. (2017)
*Ganodermagibbosum*	XSD34	EU273513	China	Retrieve from GenBank
*Ganodermahoehnelianum*	Dai12096	KU219989	China	Song et al. (2016)
*Ganodermahoehnelianum*	Yuan 6337	MG279160	China	Xing et al. (2018)
*Ganodermaleucocontextum*	GDGM44303	KJ027607	China	Li et al. (2015)
*Ganodermalingzhi*	Cui9166	KJ143907	China	Cao et al. (2012)
*Ganodermalingzhi*	Dai12574	KJ143908	China	Cao et al. (2012)
*Ganodermalingzhi*	HKAS-76642 (T)	KC222318	China	Yang and Feng (2013)
*Ganodermalingzhi*	SFC20150624.06	KY364245	Korea	Jargalmaa et al. (2017)
*Ganodermalingzhi*	SFC20150630.14	KY364246	Korea	Jargalmaa et al. (2017)
*Ganodermalobatum*	GVL-36	MT232631	Mexico	Espinoza et al. (2021)
*Ganodermalucidum*	MUCL 35119	MK554779	France	Cabarroi-Hernández et al. (2019)
*Ganodermalucidum*	RYV 33217 (T)	Z37096	Norway	Smith and Sivasithamparam (2000)
*Ganodermamartinicense*	231NC	MG654182	NC, USA	Loyd et al. (2018)
*Ganodermamartinicense*	246TX	MG654185	TX, USA	Loyd et al. (2018)
*Ganodermamartinicense*	LIP SW-Mart08-55 (T)	KF963256	Martinique	Retrieve from GenBank
*Ganodermamastoporum*	PM21	JQ409361	Malasia	Retrieve from GenBank
*Ganodermamastoporum*	TNM-F0018835	JX840351	China	Wang et al. (2012)
*Ganodermameredithae*	CBS 271.88 (T)	NR_164435	USA	Vu et al. (2019)
*Ganodermamexicanum*	MUCL 49453	MK531811	Martinique	Cabarroi-Hernández et al. (2019)
*Ganodermamexicanum*	XAL D.Jarvio 143	MK531823	México	Cabarroi-Hernández et al. (2019)
*Ganodermamizoramense*	UMN-MZ4 (T)	KY643750	India	Crous et al. (2017)
*Ganodermamizoramense*	UMN-MZ5	KY643751	India	Crous et al. (2017)
*Ganodermamultipileum*	CWN04670	KJ143913	China	Retrieve from GenBank
*Ganodermamultipileum*	Dai9447	KJ143914	China	Zhou et al. 2015
*Ganodermamultiplicatum*	CC8	KU569515	Colombia	Bolaños et al. (2016)
*Ganodermamultiplicatum*	URM 83346	JX310823	Brazil	de Lima et al. (2014)
*Ganodermaoerstedii*	GA-24	OQ845469	Costa Rica	This study
*Ganodermaoerstedii*	5191	OQ845467	FL, USA	This study
*Ganodermaoerstedii*	FDACS-DPI 2019-100390	OQ845468	FL, USA	This study
*Ganodermaorbiforme*	Cui 13880	MG279187	China	Sun et al. (2022)
*Ganodermaorbiforme*	Cui 13891	MZ354953	China	Sun et al. (2022)
*Ganodermaorbiforme*	Cui 18301	MZ354954	China	Sun et al. (2022)
*Ganodermaorbiforme*	Cui 18302	MZ354955	China	Sun et al. (2022)
*Ganodermaorbiforme*	Cui 18317	MZ354956	China	Sun et al. (2022)
*Ganodermaorbiforme*	Cui 18326	MZ354957	China	Sun et al. (2022)
*Ganodermaorbiforme*	URM 83332	JX310813	Brazil	de Lima et al. (2014)
*Ganodermaorbiforme*	URM 83334	JX310814	Brazil	de Lima et al. (2014)
*Ganodermaorbiforme*	URM 83335	JX310815	Brazil	de Lima et al. (2014)
*Ganodermaorbiforme*	URM 83336	JX310816	Brazil	de Lima et al. (2014)
*Ganodermaoregonense*	CBS 265.88	JQ781875	OR, USA	Cao et al. (2012)
*Ganodermaoregonense*	CBS 266.88	JQ781876	WA, USA	Cao et al. (2012)
*Ganodermaparvulum*	GA-04	OQ845470	Costa Rica	This study
*Ganodermaparvulum*	GA-08	OQ845471	Costa Rica	This study
*Ganodermaparvulum*	GA-09	OQ845472	Costa Rica	This study
*Ganodermaparvulum*	GA-10	OQ845473	Costa Rica	This study
*Ganodermaparvulum*	GA-46	OQ845474	Costa Rica	This study
*Ganodermaparvulum*	GA-56	OQ845475	Costa Rica	This study
*Ganodermaparvulum*	INB E.Fletes-7619	MK531821	Costa Rica	Cabarroi-Hernández et al. (2019)
*Ganodermaparvulum*	MUCL 43863	MK554769	Cuba	Cabarroi-Hernández et al. (2019)
*Ganodermaparvulum*	MUCL 44148	MK531132	Cuba	Cabarroi-Hernández et al. (2019)
*Ganodermaparvulum*	MUCL 52655	MK554770	French Guiana	Cabarroi-Hernández et al. (2019)
*Ganodermaparvulum*	MUCL53123	MK531814	French Guiana	Cabarroi-Hernández et al. (2019)
*Ganodermaphilippii*	E7092	AJ608710	Indonesia	Retrieve from GenBank
*Ganodermaphilippii*	E7098	AJ536662.2	Indonesia	Retrieve from GenBank
*Ganodermapodocarpense*	JV 1504/126	MZ354942	Costa Rica	Sun et al. (2022)
*Ganodermapodocarpense*	QCAM6422 (T)	MF796661	Ecuador	Crous et al. (2017)
*Ganodermapolychromum*	330OR	MG654196	OR, USA	Loyd et al. (2018)
*Ganodermapolychromum*	BJ280CA	MG910492	CA, USA	Loyd et al. (2018)
*Ganodermaresinaceum*	URM 83400	JX310824	Brazil	de Lima et al. (2014)
*Ganodermaresinaceum*	BR 4150	KJ143915	France	Zhou et al. 2015
*Ganodermaresinaceum*	MUCL 38956	MK554772	Netherlands	Cabarroi-Hernández et al. (2019)
*Ganodermaresinaceum*	MUCL 52253	MK554786	France	Cabarroi-Hernández et al. (2019)
*Ganodermaryvardenii*	HKAS58053 (T)	HM138671	Cameroon	Kinge and Mih (2011)
*Ganodermasessile*	MUCL 38061	MK554778	USA	Cabarroi-Hernández et al. (2019)
*Ganodermasessile*	UMNFL10	MG654227	FL, USA	Loyd et al. (2018)
*Ganodermasessile*	UMNMI24	MG654271	MI, USA	Loyd et al. (2018)
*Ganodermasichuanense*	HMAS 42798 (T)	JQ781877	China	Zhou et al. 2015
*Ganodermasinense*	Wei5327	KF494998	China	Costa-Rezende et al. (2017)
*Ganoderma* sp.	JMCR128	AF255148	Costa Rica	Moncalvo and Buchanan (2008)
*Ganoderma* sp.	JMCR132	AF255137	Costa Rica	Moncalvo and Buchanan (2008)
*Ganoderma* sp.	JMCR142	AF255138	Costa Rica	Moncalvo and Buchanan (2008)
*Ganoderma* sp.	JMCR25	AF255134	Costa Rica	Moncalvo and Buchanan (2008)
*Ganoderma* sp.	JMCR41	AF255135	Costa Rica	Moncalvo and Buchanan (2008)
*Ganoderma* sp	JMCR55	AF255136	Costa Rica	Moncalvo and Buchanan (2008)
*Ganoderma* sp.	VPB202	KJ832060	Brazil	Martin et al. (2015)
*Ganoderma* sp.	GA-27	OQ845476	Costa Rica	This study
*Ganodermasteyaertanum*	MEL2382783	KP012964	Australia	Retrieve from GenBank
*Ganodermastipitatum*	CM-UDEA110	MT945605	Colombia	Jaramillo et al. (2020)
*Ganodermasubamboinense*	Ule.2748/F 15183 (T)	MK531824	Brazil	Cabarroi-Hernández et al. (2019)
Ganodermasubamboinensevar.laevisporum	UMNFL100	MG654373	FL, USA	Loyd et al. (2018)
Ganodermasubamboinensevar.laevisporum	UMNFL32	MG654372	FL, USA	Loyd et al. (2018)
*Ganodermasubfornicatum*	BRFM 1024	JX082352	French Guiana	Berrin et al. (2012)
*Ganodermatornatum*	GVL-05	MT232633	Mexico	Espinoza et al. (2021)
*Ganodermatornatum*	URM82776	JQ514110	Brazil	de Lima et al. (2014)
*Ganodermatropicum*	KUMCC 18–0046	MH823539	Thailand	Luangharn et al. (2019)
*Ganodermatropicum*	Yuan3490	JQ781880	China	Cao et al. (2012)
*Ganodermatsugae*	Dai 12760 (IFP)	KJ143920	USA	Zhou et al. 2015
*Ganodermatsugae*	UMNMI20	MG654324	MI, USA	Loyd et al. (2018)
*Ganodermatuberculosum*	GVL-40	MT232634	Mexico	Espinoza et al. (2021)
*Ganodermatuberculosum*	PLM684	MG654369	FL, USA	Loyd et al. (2018)
*Ganodermatuberculosum*	Dai 17412	MZ354943	Brazil	Sun et al. (2022)
*Ganodermatuberculosum*	JV 1607/62	MZ354944	Costa Rica	Sun et al. (2022)
*Ganodermaweberianum*	B18	JN637827	Cuba	Torres-Farradá et al. (2016)
*Ganodermaweberianum*	CBS 1285.81	MK603805	Taiwan	Cabarroi-Hernández et al. (2019)
*Ganodermaweberianum*	CBS 219.36	MK603804	Philippines	Cabarroi-Hernández et al. (2019)
*Ganodermaweberianum*	Guzmán–Dávalos 9569	MK554771	México	Cabarroi-Hernández et al. (2019)
*Ganodermawiiroense*	UMN20GHA (T)	KT952363	Ghana	Crous et al. (2015)
*Ganodermawiiroense*	UMN21GHA (T)	KT952361	Ghana	Crous et al. (2015)
*Ganodermazonatum*	FDACS-DPI 2019-102200	OQ845478	FL, USA	This study
*Ganodermazonatum*	UMNFL105	MG654408	FL, USA	Loyd et al. (2018)
*Ganodermazonatum*	UMNFL85	MG654402	FL, USA	Loyd et al. (2018)
*Ganodermazonatum*	FDACS-DPI 2021-107113	OQ845477	FL, USA	This study
*Tomophaguscolossus* (outgroup)	TC02	KJ143923	Vietnam	Zhou et al. 2015
*Tomophaguscolossus* (outgroup)	URM80450	JX310825	Brazil	de Lima et al. (2014)

Sequence assembly and editing were performed in GENEIOUS v. 11.1.5 (Kearse et al. 2012). Alignments for each gene and both datasets were generated using MAFFT v.7.490 (Katoh and Standley 2013) with the L-INS-i algorithm. The software GBLOCKS v.0.91b (Talavera and Castresana 2007) was used to remove poorly-aligned positions and divergent regions from the DNA alignments with parameters for a less stringent selection.

PARTITION FINDER v.2.1 (Lanfear et al. 2016), implemented in the CIPRES Science Gateway web portal (http://www.phylo.org/sub_sections/portal/), following the Akaike Information Criterion (AIC), was used to select the best-fit model of evolution and the GTR+G model was applied.

Bayesian Inference (BI) and Maximum Likelihood (ML) phylogenetic analyses were applied to the dataset. The ML analysis was carried out in RAxML v.8.2.12 (Stamatakis 2014) implemented in the CIPRES Science Gateway web portal (http://www.phylo.org/sub_sections/portal/), with 1,000 non-parametric bootstrap iterations using the GTRGAMMA model and discrete gamma distribution. Bayesian analysis was performed with the programme MrBayes v.3.2.7a (Ronquist et al. 2012) on XSEDE (Miller et al. 2010) on the CIPRES Science Gateway web portal. Two parallel runs with eight chains of Metropolis-coupled Markov Chain Monte Carlo (MC)^3^ iterations were performed. Analysis was run for 100 million generations, with trees sampled every 1000^th^ generation. Burn-ins were determined by checking the likelihood trace plots in Tracer v.1.7 (Rambaut et al. 2018) and subsequently discarded. To confirm the convergence of trees, the average standard deviation of split frequencies was monitored to ensure that it fell below 0.01 and log files from the Bayesian analyses were analysed with Tracer. No indication of a lack of convergence was detected. Bayesian posterior probabilities (BPP) ≥ 0.95 and Bootstrap values (BS) ≥ 70 were considered significant. The final alignment and the phylogenetic trees are given as Suppl. materials.

## ﻿Results

### ﻿Molecular phylogeny

**Figure 1.**
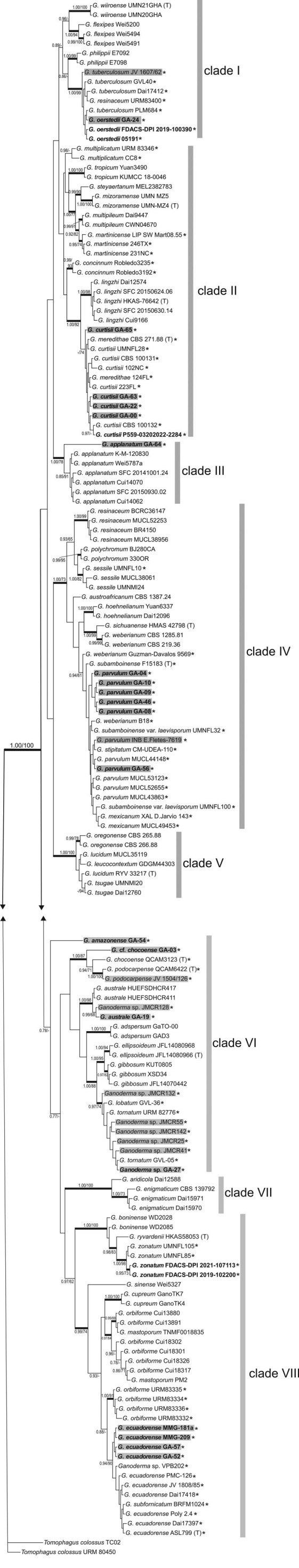
Phylogenetic tree of *Ganoderma* inferred from a Bayesian analysis, based on ITS sequence data. Bayesian posterior probabilities (BPP) > 0.84 and Maximum Likelihood Bootstrap scores (BS) > 70% are shown at the nodes at the first and second positions. BPP ≥ 0.95 and BS ≥ 70 were significant and are indicated by thickened branches. The phylogenetic position of the species occurring in Costa Rica is highlighted in grey. Sequences generated in this study are shown in bold. The (T) indicates type material and the asterisk (*) indicates specimens from sub-neotropical and neotropical regions.

A total of 25 ITS sequences were generated from eight neotropical species of *Ganoderma* that were aligned with other 62 congenetic species. The dataset contained 159 sequences and 465 base pairs in length. The BI and ML phylogeny showed similar tree topologies with *Ganoderma* as a robust monophyletic clade (1/100) comprising eight core clades (I to VIII) including 42 terminal clades that varied in terms of support (Fig. 1, only the BI tree is shown).

The sister-group relationships amongst these eight clades remained with low to moderate support (average BPP: 0.58). On the other hand, the support of several terminal clades, which may represent the circumscription of species, was moderate to strong in most terminal branches (average BPP: 0.96). The sequences obtained from Costa Rican specimens clustered in six of the eight clades (except V and VII).

Clade I is a weakly-supported clade (0.89/34) and included sequences labelled as *G.wiiroense* E.C. Otto, Blanchette, C.W. Barnes & Held from Ghana, *G.flexipes* Pat. from China, *G.philippii* (Bres. & Henn. ex Sacc.) Bres. from Indonesia and China and *G.tuberculosum* - *G.oerstedii* from Brazil, Mexico and the USA. The sequences from specimens collected in Costa Rica GA-24 and JV-1607/62 clustered with sequences of *G.tuberculosum* and *G.oerstedii*, forming a well-supported monophyletic group (1/99). Within this clade, where species were represented by more than two sequences, the terminal clades were strongly supported, i.e., *G.flexipes* (1/94), *G.philippi* (1/99) and *G.wiroense* (1/100).

Clade II is divided into two major subclades (0.61/40): clade II.A contains sequences of non-laccate species labelled as *G.multiplicatum* from Brazil and Colombia, *G.tropicum* from China and Thailand, *G.steyaertanum* from Australia, *G.mizoramense* Zothanz., Blanchette, Held & C.W. Barnes from India, *G.multipileum* from China and *G.martinicense* from Martinique and southern USA. The support for this subclade and the internal relationships amongst the species were weak (0.81/54). Clade II.B, resolved with strong support (1/92) and is divided into two subclades: one with sequences labelled as *G.lingzhi* from China and Korea (1/98) and another one with sequences named as *G.curtisii* and *G.meredithae* Adask. & Gilb. (including the type) from North America and the sequences of the Costa Rican specimens GA-00, GA-22, GA-63 and GA-65 (0.58/74).

Clade III grouped sequences tagged as *G.applanatum* (1/78). The vouchers of several collections in China and UK were grouped together and the sequence obtained from the Costa Rican specimen GA-64 formed an independent lineage.

Clade IV was divided into two subclades (1/73). Clade IV.A contained sequences labelled as *G.resinaceum* from Europe and *G.sessile* Murrill and *G.polychromum* (Copel.) Murrill from the USA (0.93/65). Clade IV.B was divided into two subclades (0.94/61): Clade IV.B.1 grouped sequences of *G.hoehnelianum* Bres., *G.sichuanense* and *G.weberianum* from East Asia and Clade IV.B.2 with sequences of *G.mexicanum*, *G.parvulum*, *G.stipitatum* and *G.subamboinense* (Henn.) Bazzalo & J.E. Wright from the Neotropics. Sequences of seven specimens of *G.parvulum* from Costa Rica (Fletes 7619, GA-04, GA-08, GA-09, GA-10, GA-46 and GA-56) were placed within this subclade.

Clade VI was a weakly supported clade (0.66/16) that contained several non-laccate species. This clade was divided into three subclades. Clade VI.A (1/87) with sequences of *G.chocoense* and *G.podocarpense* from Ecuador (including type specimens) and the Costa Rican specimen GA-03. Clade VI.B (1/98) with sequences of *G.australe* from Australia and the Costa Rican specimens JMCR-128 and GA-19. Clade VI.C was subdivided into three terminal clades with strong support. Clade VI.C.1 with sequences from *G.adspersum* (Schulzer) Donk from Europe (1/100), Clade VI.C.2 that groups sequences of *G.gibbosum* (Blume & T. Nees) Pat. and *G.ellipsoideum* Hapuar., T.C. Wen & K.D. Hyde from East Asia (0.97/82) and Clade VI.C.3 with several sequences from the Neotropics (0.97/74), including vouchers labelled as *G.lobatum* (Cooke) G.F. Atk. and *G.tornatum* (Pers.) Bres. from Brazil and Mexico and several unidentified specimens from Costa Rica (JMCR25, JMCR55, JMCR142, JMCR41, JMCR132 and GA-27). A single sequence from the Costa Rican specimen GA-54, identified as *G.amazonense*, was grouped within this clade with low support as an independent lineage in both phylogenies (0.73/23).

Clade VIII was divided into two strongly-supported subclades (0.97/62). Clade VIII.A that grouped sequences of *G.boninense* Pat. from Japan, *G.ryvardenii* Tonjock & Mih from Cameroon and *G.zonatum* Murrill from Florida (USA) (1/100). Clade VIII.B (0.99/74) was divided into two poorly-supported subclades: Clade VIII.B.1 that included sequences labelled as *G.sinense* J.D. Zhao, L.W. Hsu & X.Q. Zhang from China, *G.cupreum* from Cameroon, *G.mastoporum* from China and Malaysia and *G.orbiforme* from China; and Clade VIII.B.2 that grouped sequences labelled as *G.orbiforme* from Brazil and *G.ecuadorense* A. Salazar, C.W. Barnes & Ordoñez from several neotropical countries. Four sequences from Costa Rican specimens (MMG-181a, MMG-209, GA-57, GA-52) were placed within a well-supported terminal clade (0.94/90) with sequences of *G.ecuadorense* from Brazil, Ecuador and French Guyana, including the type specimen.

### ﻿Identification of *Ganoderma* collections

**Figure 2.**
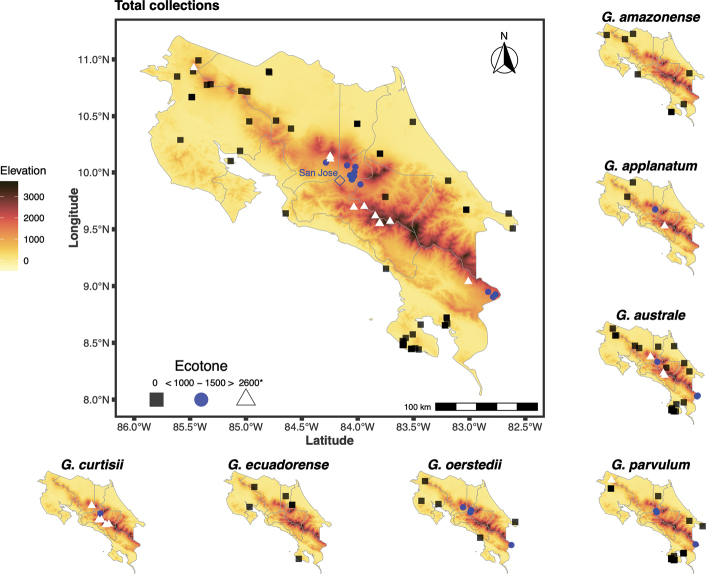
Distribution map of the seven *Ganoderma* species from Costa Rica.

In this study, 117 specimens of *Ganoderma* were studied in detail. Collections originated from all over the country. Seven taxa were identified: *G.amazonense* (n = 9), *G.applanatum* (n = 5), *G.australe* (n = 31), *G.curtisii* (n = 15), *G.ecuadorense* (n = 9), *G.oerstedii* (n = 10) and *G.parvulum* (n = 24). The following type specimens were examined: *Fomesstipitatus* Murr., *Ganodermaamazonense* Weir, *G.dorsale* (Lloyd) Torrend, *G.oerstedii* (Fr.) Torrend, *G.perzonatum* Murrill, *G.pulverulentum* Murrill, *G.sessile* Murrill, *G.sessiliforme* Murrill and G. *tuberculosum* Murrill. We also include a map showing the distribution of *Ganoderma* in Costa Rica, based on the altitudinal gradient in Costa Rica and the location of the studied vouchers (Fig. 2).

### ﻿Taxonomy

**Figure 3.**
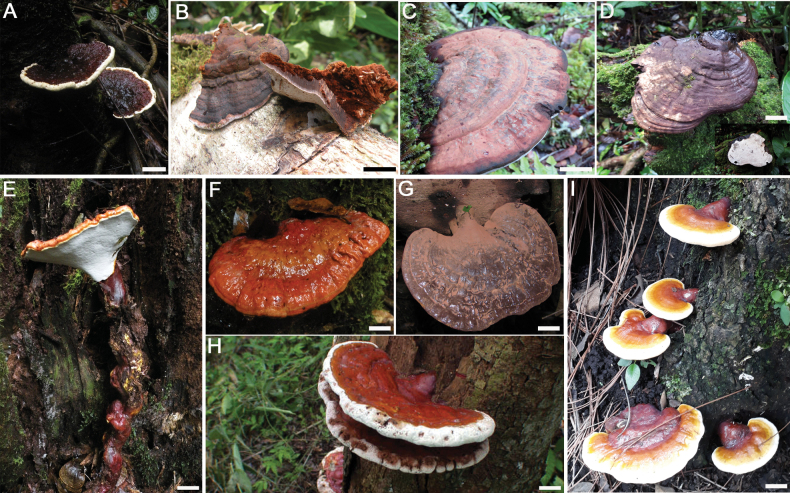
In-situ photos of basidiocarps of *Ganoderma* spp. in Costa Rica **A***G.amazonense* (GA-30) **B***G.applanatum* (GA-54) **C***G.australe* (GA-58) **D**G.cf.chocoense (GA-03) **E, F***G.curtisii* (JCV 128-10) **G***G.ecuadorense* (MMG-181) **H***G.oerstedii* (Saenz 2049) **I***G.parvulum* (GA-09). Scale bars: 20 cm (**A, H**); 3 cm (**B, C**); 1 cm (**D, E, I**).

Based on the phylogenetic relationships, morphological characteristics and geographic distribution, the *Ganoderma* specimens collected from Costa Rica were identified as: *G.amazonense*, *G.applanatum* s.l., *G.australe* s.l, *G.curtisii*, *G.ecuadorense*, *G.oerstedii* and *G.parvulum* (Fig. 3). The detail morphological descriptions of seven species, as well as important information about two doubtful species: G.applanatumvar.laevisporum C.J. Humphrey & Leus-Palo and *G.chocoense*, are provided.

#### ﻿1. Ganoderma amazonense

Taxon classificationFungiPolyporalesPolyporaceae

Weir, A pathological survey of the para rubber tree (Hevea brasiliensis Müll. Arg.) in the Amazon Valley: 12 (1926).

0CBF23C6-0251-52D6-817A-D32FC1EA9FA0

Figs 3A , 4

##### Type.

Brazil. Amazonas: Cocal Grande, Para, on *Heveabrasiliensis* (Willd. ex A.Juss.) Müll.Arg., 20 Aug 1923, James R. Weir. Pathological & Mycol. *s.n.* (lectotype: BPI62043!).

##### Description.

***Basidiocarps*** perennial, pileate, stipitate, sessile or with a contracted lateral base, corky to woody, solitary, applanate, irregular to tuberculate, 8.5 × 11 × 1 cm; ***pileus*** surface sulcate, glabrous, dull, brownish-grey to reddish-brown azonate or with zones close to the margin, margin obtuse, yellowish-brown; ***context*** yellowish-white, without resinous deposits or with fine discontinue light brown horizontal bands; ***pore surface*** pinkish-brown to yellowish-brown, pores circular 4–6 per mm; ***tube layer*** pinkish-brown to yellowish-brown, simple, up to 20 mm thick. ***Stipe*** concolour with the pileus surface, up to 5 cm long. ***Hyphal system*** dimitic; contextual generative hyphae hyaline, thin-walled, with clamps, 2–5 µm in diam., difficult to observe; skeletal hyphae thick-walled, yellowish-brown, aseptate, 3–5 µm in diam., occasionally branched. ***Cuticular cells*** from the pileus absent. ***Basidia*** not observed. ***Basidiospores*** ovoid to ellipsoid, truncate at the distal end; with two walls, connected by inter-wall pillars, hyaline to yellowish-brown, negative in Melzer’s Reagent, 8–10 × 6–7 µm. ***Chlamydospores*** not observed.

##### Descriptions and illustrations.

Weir (1926), Furtado (1967), Steyaert (1980), Gottlieb and Wright (1999a), Ryvarden (2004), Torres-Torres et al. (2012).

##### Substrata.

On hardwood logs.

##### Altitudinal distribution.

Lowlands.

##### Geographic distribution.

*G.amazonense* is reported in the Caribbean (Jamaica and Puerto Rico) and Central and South America (Costa Rica, Honduras and Brazil). Reports in West and Central Africa (Steyaert 1980) need further confirmation.

##### Specimens examined.

Costa Rica. Alajuela: Los Chiles, Reserva Nacional de Vida Silvestre Caño Negro, 10°53'6.71"N, 84°47'28.27"W, 30 m elev., 03 Aug 1991, A. Ruiz-Boyer 7-91 (USJ36351). Upala, Bijagua, Albergue Heliconias, 10°43'21.05"N, 85°2'30.47"W, 500 m elev., on log, 12 Jul 2001, L. Ryvarden 43716 (CR3802379). Guanacaste: Liberia, Parque Nacional Santa Rosa, sector Bosque Húmedo, 10°50'57.49"N, 85°36'57.89"W, 300 m elev., on log, 24 Oct 1996, I. Lindblad 2144.2 (CR3131819). Limón: Cantón Central, Reserva Biológica Hitoy Cerere, Sendero Tepezcuintle, 9°40'19.97"N, 83°01'42.96"W, 100 m elev., on log, 23 Jul 2003, E. Navarro 6843 (CR3727415). Puntarenas: Garabito, Jacó, Sector Garabú, Finca Quebrada Bonita, 9°38'22.81"N, 84°38'40.81"W, 100 m elev., on log, 24 Nov 2008, E. Navarro 10912 (CR4188987); Osa, Parque Nacional Piedras Blancas, Estación Río Bonito, Sendero Tacho, 9°38'22.81"N, 84°38'40.81"W, 100 m elev., on log, 14 Mar 2003, E. Fletes 4933 (CR3700169); Osa, Parque Nacional Corcovado, Estación Sirena, Sendero Espaveles, 8°28'57.75"N, 83°35'28.87"W, 0–10 m elev., on log, 14 Sep 2001, E. Fletes 2847 (CR3756152); 8°28'59.54"N, 83°35'29.69"W, 0–10 m elev., 14 Jul 2021, J. Carranza, M. Mardones, E. Fletes GA-30 (USJ109778); Sendero Sirena, 8°28'56.01"N, 83°35'49.16"W, 0–30 m elev., on log, 06 Jul 2022, J. Carranza, M. Mardones, E. Fletes GA-54 (USJ109779, sequence ITSOQ845454).

##### Discussion.

*Ganodermaamazonense* was described by Weir (1926) as a new species from the Amazonas (Brazil) decaying the roots of *Hevea* spp. It is characterised by the dull-brown, non-laccate pileus surface, the pale context and the small, light yellow basidiospores. The basidiospores of the specimens from Costa Rica examined in this study are ellipsoid, echinulate and truncate and measure 8–10 × 6–7 µm that agree with measurements reported by Welti and Courtecuisse (2010) and Torres-Torres et al. (2012). However, slightly smaller basidiospores have been observed in the type specimen (6–9.35 × 4–6 µm) and in descriptions by Gottlieb and Wright (1999a), Ryvarden (2004) and Gomes-Silva et al. (2011). All the specimens of *G.amazonense* examined in this study were collected in lowlands.

**Figure 4.**
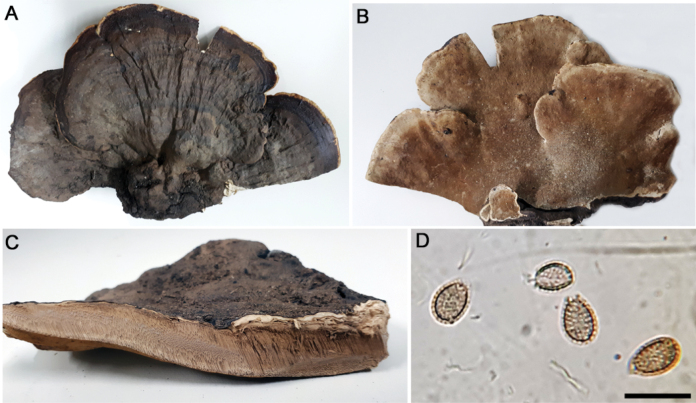
*Ganodermaamazonense***A** basidiocarp pileus (Fletes 2847) **B** pore surface (Fletes 2847) **C** context tissue (Navarro 6843) **D** basidiospores (Fletes 4933). Scale bar: 10 µm (**D**).

The *G.amazonense* sequence (GA-54) was placed in our phylogeny as a sister lineage of clade VI with moderate support in the BI analysis (0.78). Our sequence constitutes the first molecular record for this species deposited in GenBank. More sequences from additional molecular markers are needed to confirm the species’ evolutionary relationships with other *Ganoderma* species, but its position as a separate lineage within the genus is confirmed.

#### ﻿2. Ganoderma australe

Taxon classificationFungiPolyporalesPolyporaceae

(Fr.) Pat., Bull. Soc. mycol. Fr. 5(2,3): 65 (1889).

2402BAE6-9E5D-5C41-888B-1974649A9B46

Figs 3C , 5

≡ Polyporus australis Fr., Elench. fung. 1: 108 (1828).

##### Type.

An island in Pacific Ocean, on log, *s.d.*, *s.n.* (type lost).

##### Description.

***Basidiocarps*** perennial, sessile or with a contracted lateral base, dimidiate, woody, solitary, applanate to ungulate, irregular to tuberculate, 1.6–21.2 × 1.5–32 × 0.3–5.1 cm; ***pileus*** surface crustose, rugulose, sulcate, glabrous, dull, greyish-brown, yellowish-brown, reddish-brown to brownish-black, margin obtuse, yellowish-brown to pinkish-brown, azonate or with brownish-black, reddish-brown or yellowish-brown zones; ***context*** corky, vinaceous, purple-brown or yellowish-brown, with horizontal bands of melanoid substances, 1–30 mm thick, becoming dark with KOH; ***pore surface*** pinkish-brown to yellowish-brown, pores circular, 3–5 per mm; ***tube layers*** concolorous with context or yellowish-brown, sometimes whitish within, tubes simple to stratified, up to 0.5–25 mm thick. ***Hyphal system*** dimitic or trimitic; contextual generative hyphae inconspicuous, thin-walled, with clamps, hyaline, 1.5–3 µm diam.; skeletal hyphae thick-walled, yellowish-brown, aseptate, up to 6 µm in diam., occasionally branched; binding hyphae thin-walled, 1–2 µm in diam. ***Cuticular cells*** from the pileus: absent. ***Basidia*** difficult to find. ***Basidiospores*** ovoid, truncate at the distal end; with two walls, connected by inter-wall pillars, yellowish-brown, negative in Melzer’s Reagent, 7–12 × 5–8 µm. ***Chlamydospores*** not observed.

**Figure 5.**
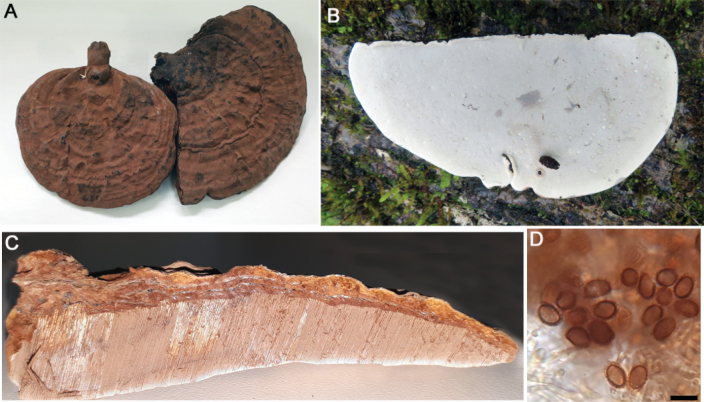
*Ganodermaaustrale***A** basidiocarp pileus (Fletes 341) **B** pore surface (GA-64) **C** context tissue (Fletes 1403) **D** basidiospores (Fletes 1403). Scale bar: 10 µm (**D**).

##### Descriptions and illustrations.

Furtado (1967), Ruiz-Boyer (1998), Ryvarden (2004), Welti and Courtecuisse (2010).

##### Substrata.

Dead-standing hardwood trees, stumps or logs.

##### Altitudinal distribution.

Lowlands to highlands.

##### Geographic distribution.

Pantropical, common in tropical America.

##### Specimens examined.

Costa Rica. Alajuela: Arenal, Parque Nacional Arenal, sendero Pilón, 10°27'39.29"N, 84°43'51.83"W, 600–700 m elev., 15 Jul 2001, A. Ruiz 521 (CR3802311); Poás, Parque Nacional Volcán Poás, Sendero hacia el Bosque del Niño, 10°7'3.27"N, 84°14'36.88"W, 2500–2600 m elev., 27 Jun 2007, E. Navarro 10184 (CR4089856); San Carlos, Pocosol, Finca Latite, 10°23'26.51"N, 84°35'49.69"W, 110 m elev., 29 May 2002, J. Carranza JCV 13-02 (USJ72910). Cartago: Jimenez, Pejibaye, Refugio de Vida Silvestre El Copal, 9°47'6.90"N, 83°45'7.77"W, 650 m elev., 26 Apr 2006, E. Navarro 9620 (CR4014312). Guanacaste: La Cruz, Parque Nacional Guanacaste, Estación Biológica Pitilla, camino a la Esperanza, 10°59'28.61"N, 85°25'33.17"W, 700–800 m elev., 23 Mar 1997, C. Cano 1012 (CR1544454); Liberia, Parque Nacional Rincón de la Vieja, Estación San Cristóbal, Sendero La Danta, 10°46'31.27"N, 85°21'0.51"W, 600–700 m elev., 28 Sep 1996, C. Cano 615 (CR144376); Sector Santa María, Los Naranjales, 10°46'53.11"N, 85°19'1.38"W, 800–900 m elev., 05 Dec 1997, C. Cano 1237 (CR3495780). Heredia: Sarapiquí, La Virgen, Estación Biológica La Selva, 10°25'56.52"N, 84°0'13.96"W, 40 m elev., on log, 06 Nov 2016, J. Carranza JCV 2-16 (USJ109687). Limón, Cantón Central, Reserva Veragua, Sendero Los Valientes, 9°55'40.63"N, 83°11'28.53"W, 200–300 m elev., 26 Jun 2009, E. Navarro 11165 (CR4222697); Reserva Biológica Hitoy Cerere, Sendero Tepezcuintle, 9°40'19.97"N, 83°01'42.96"W, 0–100 m elev., 19 Sep 2001, R. Valladares 536 (CR3464661). Pococí, Colorado, Tortuguero, Reserva Biológica del Bosque Lluvioso, 10°26'58.96"N, 83°30'25.19"W, 300–400 m elev., 29 Jan 2004, E. Alvarado 111 (CR3802764). Puntarenas: Cantón Central, Parque Nacional Isla del Coco, orillas del Río Genio, 5°30'15.64"N, 87°4'32.05"W, 0–100 m elev., 04 Jun 2005, E. Fletes 7607 (CR3976554). Coto Brus, San Vito, Parque Nacional La Amistad, Zona Protegida Las Tablas, Fila Chiquizá, 8°55'34.40"N, 82°46'00.950"W, 1500–1600 m elev., 18 Feb 2003, E. Fletes 4870 (CR3575822); Finca Cafrosa, Pizote, 8°54'15.82"N, 82°47'21.22"W, 1400–1500 m elev., 28 Nov 1998, E. Navarro 520 (CR4109271). Osa, Puerto Escondido, Playa Colibrí, 8°39'36.96"N, 83°26'12.46"W, 0–100 m elev., 5 Nov 2006, E. Alvarado 367 (CR4044781); Parque Nacional Piedras Blancas, Estación Río Bonito, sendero a San Josecito, 8°43'16.18"N, 83°12'14.64"W, 400 m elev., 18 Apr 1999, E. Fletes 341 (CR1546010); Karate, Finca Exótica, 8°26'29.64"N, 83°27'15.39"W, 0–10 m elev., 11 Aug 2019, M. Mata JCV 4-19 (USJ109489); Parque Nacional Corcovado, Estación San Pedrillo, Sendero Llorona, 8°29'1.96"N, 83°35'30.31"W, 10–100 m elev., 16 Feb 2000, E. Fletes 1219 (CR3097854); Sector Sirena, Sendero Espaveles, 8°29'3.30"N, 83°35'30.64"W, 0–100 m elev., 08 Feb 2003, E. Fletes 4860 (CR3575815); 8°28'46.91"N, 83°35'22.30"W, 0–100 m elev., 01 Jun 2012, J. Carranza JCV 310-12 (USJ109694); Sendero Ollas-Sirena, 8°29'5.14"N, 83°35'24.33"W, 0–100 m elev., 01 Jun 2012, J. Carranza JCV 42-12 (USJ109489); Sector Sirena, sendero a Río Pavo, 8°30' 23.51"N, 83°35'19.34"W, 0–100 m elev., 25 Mar 2003, E. Fletes 1403 (CR1547383); Sendero Espaveles a sendero la Olla, 8°29'4.60"N, 83°35'22.49"W, 0–30 m elev., on log, 07 Jul 2022, J. Carranza, M. Mardones, E. Fletes GA-58 (USJ109795); Sector Aguas Azules, 8°32'35.08"N, 83°34'13.43"W, 0–100 m elev., 12 Mar 2005, E. Fletes 7302 (CR3994940); Estación La Leona, Sendero Paraíso, 8°26'50.34"N, 83°31'6.19"W, 0–100 m elev., 10 Sep 2009, J. Carranza JCV 25-09 (USJ109489); 8°26'49.55"N, 83°31'8.89"W, 0–100 m elev., 9 Dec 2016, J. Carranza JCV 8-16 (USJ109686); 8°26' 50.79"N, 83°31'14.79"W, 0–100 m elev., 08 Jan 2009, J. Carranza JCV 104-09 (USJ109489). San José, Dota, Reserva Forestal Los Santos, Albergue de Montaña Savegre, Sendero Los Robles, 9°33'00.00"N, 83°48'00.0"W, 2400–2500 m elev., 20 Jun 2005, R. Rodríguez 505 (CR3968596); Finca La Neblina, sendero de las Torres a Savegre, 9°37'3.65"N, 83°50'33.3"W, 2500–2600 m elev., 14 Oct 2006, E. Navarro 99712 (CR4043836); Cerro de la Muerte, Km 92.5, Estación Los Nímbulos, sendero en el robledal, 10°25'18.9"N, 84°01'30.6"W, 3100 m elev., 09 Jun 2019, M. Mardones GA-19 (USJ109713, sequences ITSOQ845456, LSUOQ835180). Moravia, Jardínes, 9°58'1.31"N, 84°1'58.2"W, 1300 m elev., 12 Sep 2021, J. Carranza JCV 2-21 (USJ109781).

##### Discussion.

*Ganodermaaustrale* is a common species in the Tropics that traditionally is considered a cosmopolitan species; but recent studies suggest that *G.australe* is only present in America and Oceania (Fryssouli et al. 2020). Macroscopically, the main characteristics of *G.australe* are tough and sessile basidiocarp with distinct black cuticle, greyish to brown pileus and context with resinous deposits or melanoid bands. Microscopically can be recognised by its cylindrical and hyaline basidiospores.

The Costa Rican specimens have a wide range of colour variations of the pileus and spore sizes. Steyaert, cited by Ryvarden and Johansen (1980), reported spore sizes that range from 6–13 × 4.5–8 µm, while Ruiz Boyer (1998) found 6–8 × 4–6 µm and Ryvarden (2004) mentioned spore sizes of 7–12 × 5–8 µm. The spore sizes of the specimens observed in our study were in the range of the ones mentioned by these authors. Morphologically, amongst the neotropical species of *Ganoderma* with non-laccate basidiocarps, *G.australe* and *G.applanatum* are difficult to differentiate. However, both species can be distinguished by the resinous deposits or melanoid bands present only in the context of *G.australe*. From the morphological examination of ca. 40 herbarium specimens within the *G.applanatum-australe* complex in Costa Rica, we determined that most specimens belong to *G.australe*, with a few occurrences of *G.applanatum* (see below). There are some specimens of *G.australe* that do not show resinous deposits or melanoid bands or are very inconspicuous. In these cases, the size of the spores (larger in *G.australe* than in *G.applanatum*) is a criterion to distinguish both species. In other cases, the morphological distinction is complex and molecular characterisation should be used.

Identifying *G.australe* using the ITS region is challenging since, according to Fryssouli et al. (2020), about 5% of the *Ganoderma* sequences deposited in GenBank are labelled as *G.australe.* Still, only 22% of them are correctly tagged. We selected two reference sequences of *G.australe* from Australia (DHCR411 and DHCR417) to be included in the phylogeny. The sequences JMCR128 and GA-19 grouped with them in a strongly supported subclade (1/98) within clade VI.

#### ﻿3. Ganoderma applanatum

Taxon classificationFungiPolyporalesPolyporaceae

(Pers.) Pat., Hyménomyc. Eur. (Paris): 143 (1887)

2692DFFF-0A61-583D-A433-9DEBAE6376F0

Fig. 3B

≡ Boletus applanatus Pers., Obs. Mycol. 2:2. 1799.

##### Description.

***Basidiocarps*** perennial, sessile or with a contracted lateral base, dimidiate, woody, solitary, applanate to ungulate, irregular to tuberculate, 2–13 × 2–22 × 0.5–10 cm; ***pileus*** surface rugulose, glabrous, dull, greyish-brown to black, margin obtuse, zonate, whitish; ***context*** firm, reddish-brown, 10–50 mm thick, becoming dark with KOH; ***pore surface*** light brown to yellowish-brown, pores circular, 4–6 per mm; ***tube layers*** concolorous with context or yellowish-brown, up to 40 mm thick. ***Hyphal system*** dimitic or trimitic; contextual generative hyphae thin-walled, with clamps, hyaline, 2–4 µm diam.; skeletal hyphae thick-walled, yellowish-brown, aseptate, 2–4 µm diam., branched; binding hyphae thick-walled, branched, hyaline, 1–2 µm diam. ***Cuticular cells*** from the pileus: absent. ***Basidia*** not observed. ***Basidiospores*** ovoid, truncate; with two walls, yellow, negative in Melzer’s Reagent, 7–10 × 5–6 µm. ***Chlamydospores*** not observed.

##### Descriptions and illustrations.

Gilbertson and Ryvarden (1986), Ruiz-Boyer (1998).

##### Substrata.

Dead-standing hardwood trees or logs.

##### Altitudinal distribution.

Lowlands to highlands.

##### Geographic distribution.

Pantropical, common in tropical America.

##### Specimens examined.

Costa Rica. Alajuela: Los Chiles, Refugio Nacional de Vida Silvestre Caño Negro, 10°53'36.73"N, 84°47'45.49"W, 10 m elev., 07 Sep 1991, A. Ruiz-Boyer 13-91 (USJ36357). Guanacaste: Tilarán, 10°27'13.66"N, 84°58'13.61"W, 534 m elev., 10 Oct 1980, J. A. Saénz & J. Carranza 314-80 (USJ21274). Heredia: Bosque de La Hoja, 10°3'44.38"N, 84°5'43.09"W, 1496 m elev., 05 Mar 1986, J. Carranza JCV 67-86 (USJ22291). San José: Dota, San Gerardo, 9°33'1.63"N, 83°48'9.66"W, 2000–2300 m elev., 18 Sep 2022, M. Mardones GA-64 (USJ109782, sequences ITSOQ845455, LSUOQ835179); El Empalme, Ojo de agua, 2250 m elev., 28 Oct 1979, J. Carranza JCV 131-79 (USJ21297).

##### Discussion.

As mentioned above, the species *G.applanatum* is morphologically similar to *G.australe*, but the shorter basidiospores and the absence of resinous deposits or melanoid substances in the context of *G.applanatum* can distinguish them. According to Ryvarden (2004), *G.applanatum* is a species restricted to temperate zones and, according to Fryssouli et al. (2020), it has a Holarctic distribution. However, our results show the presence of this species or a species closely related to *G.applanatum*, in Costa Rica. The sequence from the GA-64 specimen clusters, as an independent lineage, with several sequences identified as *G.applanatum* from Europe and Asia in a strongly-supported terminal clade (1/78) within clade III. This result would be the first record of this species in the Tropics confirmed by molecular data. Therefore, considering the morphological examination and the phylogenetic position of the sequence, we have decided to identify this specimen as *G.applanatum*. Increasing the number of collections and molecular data is essential to determine if the species observed in Costa Rica is *G.applanatum* or a closely-related species.

While examining the *G.applanatum* specimens from Costa Rica, we found four specimens with smooth basidiospores, which agree with the description of G.applanatumvar.laevisporum C.J. Humphrey & Leus-Palo. For details on these specimens, see the Excluded Species section below.

#### ﻿4. Ganoderma curtisii

Taxon classificationFungiPolyporalesPolyporaceae

(Berk.) Murrill, N. Amer. Fl. (New York) 9(2): 120 (1908).

23D249AE-3568-56DC-9DD5-0CB335049CCA

Figs 3E, F , 6

≡ Polyporus curtisii Berk. 1849.

##### Type.

USA, South Carolina, *s.d.*, *s.n.* (type: PH00042681).

##### Descriptions.

***Basidiocarps*** solitary, laterally and long stipitate, reniform, dimidiate or circular, 10.5–11.1 × 6.3–9.9 × 0.7–2.5 cm; ***pileus*** single or several arising from a branching stipe, cespitose, glabrous, shiny both when fresh and dry, laccate, upper surface yellow, yellowish-brown to reddish-brown with purple hues; ***context*** firm, buff to light brown, duplex, without concentric growth zones, 7–13 mm thick, with continuous melanoid bands embedded in context tissue, originating from the stipe and running parallel to the upper surface; ***pore surface*** pinkish-brown to yellowish, darkening when handled, pores circular to irregular, 4–6 per mm; ***tube layers*** ochraceous-tawny, 10–12 mm thick. ***Stipe*** lateral, 30–250 mm long, round, or slightly compressed, 12–18 mm diam. and with a purple to black, shiny cuticle. ***Hyphal system*** trimitic; contextual generative hyphae thick-walled, with clamps, hyaline, 3.5 µm in diam.; skeletal hyphae thick-walled, 1.5–6 µm in diam., light yellow; binding hyphae thin and thick-walled, 3–5 µm in diam. ***Cuticular cells*** from the pileus clavate, some nodulose, sometimes with 1 to 2 protuberances, rarely branched, with granulations in the apex, yellowish, with strong amyloid reaction with Melzer’s Reagent, 45–55 × 9–14 µm. ***Basidia*** not observed. ***Basidiospores*** ellipsoid to oblong, truncate at the distal end; with two walls, yellowish-brown to brown, moderately coarsely echinulate, (9–)11–17 × (7–)8–10 µm. ***Chlamydospores*** not observed.

**Figure 6.**
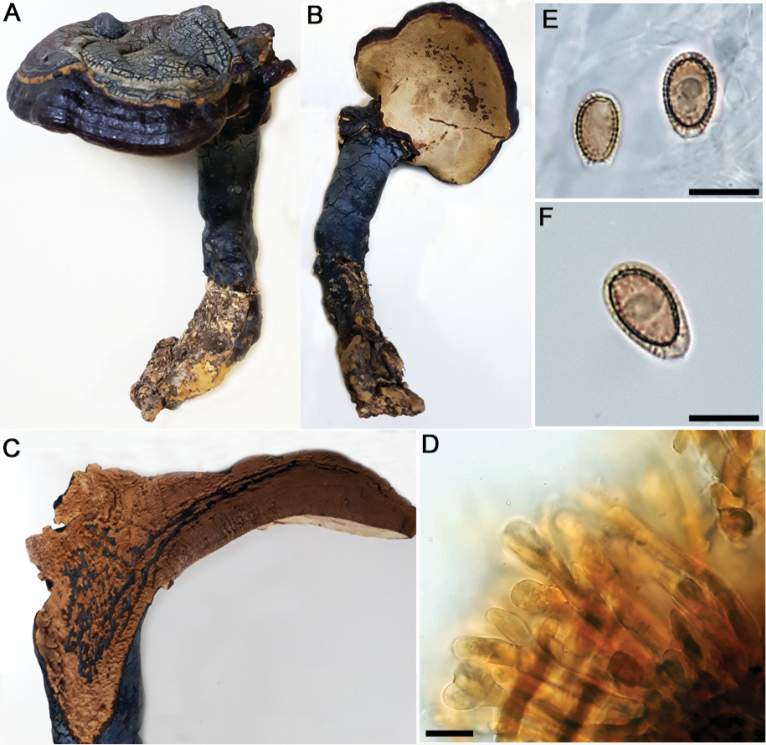
*Ganodermacurtisii***A, B** basidiocarp (Navarro 10257) **C** context tissue (Navarro 10257) **D** cuticular cells (Navarro 10257) **E, F** basidiospores (Navarro 10132). Scale bars: 10 µm (**D–F**).

##### Descriptions and illustrations.

Torres-Torres and Guzmán-Dávalos (2005, 2012), Lopez-Peña et al. (2016).

##### Substrata.

On *Quercus* spp. or *Pinus* spp., on decaying wood.

##### Altitudinal distribution.

In Costa Rica, this species is found only in the highlands.

##### Geographic distribution.

Mexico and the USA. This is the first report in Costa Rica and Central America.

##### Specimens examined.

Costa Rica. Alajuela: Grecia, Reserva Forestal Grecia, Bosque del Niño, sendero al acueducto, 10°8'30.90"N, 84°14'49.39"W, 1800–1900 m elev., 26 Jun 2006, E. Navarro 10132 (CR4089789); on soil, 10 Jul 2016, M. Mata 2647 (USJ109166). Cartago: Paraíso, Reserva Forestal Río Macho, Villa Mills, finca Los Abarca, 31 Aug 2008, 9°34'11.15"N, 83°42'37.40"W, 2600–2700 m elev., E. Alvarado 417 (CR4164678); Sector La Chonta, km. 55 de la carretera Interamericana Sur, 9°42'00.0"N, 83°56' 30.0"W, 2400–2500 m elev., 20 Jul 2007, E. Navarro 10257 (CR4101818); La Unión, Tres Ríos, Zona Protectora de La Carpintera, 9°53'44.38"N, 83°58'31.79"W, 1400 m elev., 2014, Alvarenga and Canessa GA-00 (USJ109783, sequences ITSOQ845458, LSUOQ835182). San José, Desamparados, San Miguel, Jericó, Cerro Tablazo, ladera SO, *Quercus* sp. forest, 9°49'24.34"N, 84°2'26.56"W, 1880 m elev., on log, 30 Mar 2010, Carlos O. Morales s.n. (USJ83642). Dota, San Gerardo, 9°33'0.86"N, 83°48'16.20"W, 2000–2300 m elev., 10 Jul 2000, R. Halling s.n. (USJ 71604); 9°32'59.91"N, 83°48'18.26"W, 2300 m elev., 26 Nov 2010, J. Carranza JCV 128-10 (USJ104499); 9°33'1.13"N, 83°48'22.39"W, 2300 m elev., 10 Feb 2011, J. Carranza JCV 146-11 (USJ109500); 9°33'2.08"N, 83°48'26.31"W, 2200 m elev., 18 Sep 2022, M. Mardones GA-65 (USJ109784, sequences ITSOQ845461, LSUOQ835184); 9°33'3.85"N, 83°48'25.63"W, 2200 m elev., 18 Sep 2022, M. Mardones GA-63 (USJ109785, sequences ITSOQ845460, LSUOQ835183); Santa María, Jardín, 9°43'20.15"N, 83°58'28.91"W, 2200 m elev., 28 Oct 1979, J. Carranza JCV 90-79 (USJ21299). León Cortés, San Pablo, Sector el casquillo, forest of *Quercus* spp., 9°41'37.98"N, 84°2'6.03"W, 2100 m elev., 22 Sep 2019, Beatriz Picado BPH16/GA-22 (USJ109794, sequences ITSOQ845459). Perez Zeledón, Siberia, 9°32'49.12"N, 83°42'48.29"W, 2900 m elev., on log, José Murillo 10 (USJ109055). San Marcos, Tarrazú, Canet, 9°41'38.92"N, 84°2'5.08"W, 2200 m elev., 22 Jan 2018, Beatriz Picado BPH21 (USJ109716).

##### Discussion.

*Ganodermacurtisii* mainly differs from other *Ganoderma* species from Costa Rica by its lateral and long stipe, the colour of the stipe and pileus, the melanoid bands that originate from the stipe and run parallel to the upper surface of the context and the large basidiospores (11–17 × 8–10 µm). The Costa Rican specimens examined by us showed larger basidiospores than those reported by Murrill (1915, 9–11 × 5–8 µm), Torres-Torres and Guzmán-Dávalos (2005, 10.4–12.8 × 5.6–8 µm) and Loyd et al. (2018, 8.3–12.1 × 5.4–7.5 µm). Additionally, the cuticular cells in our specimens have a very strong amyloid reaction not mentioned by Torres-Torres and Guzmán-Dávalos (2005).

In Costa Rica, this species has been found in highlands and always associated with decaying wood in *Quercus* or *Pinus* forests. Torres-Torres and Guzmán-Dávalos (2012) reported it in Mexico occurring in the same type of forests. Ganodermacurtisiif.sp.meredithiae was recently erected to include those forms characterised by occurring exclusively on pines and showed slow cultural growth rate (Loyd et al. 2018). Amongst the examined Costa Rican specimens, only one (GA-00) occurred in a pine forest; the other specimens were found in *Quercus* forests. Sequences from four specimens of *G.curtisii* from Costa Rica (GA-00, GA-22, GA-63 and GA-65) clustered in the same clade with *G.lingzhi* (clade II) with strong support (1/92), forming a terminal subclade with sequences labelled as *G.curtisii* and *G.meredithae* from the USA. This is the first report of the species in Central America and its distribution is probably strongly linked to the distribution of its host plants.

#### ﻿5. Ganoderma ecuadorense

Taxon classificationFungiPolyporalesPolyporaceae

A. Salazar, C.W. Barnes & Ordoñez [as ‘ecuadoriense’], in Salazar, Ordoñez, Toapanta, Barnes & Gamboa, Persoonia 36: 441 (2016)

B2A8E0CA-E089-5047-9158-D4F768E279BF

Figs 3G , 7

##### Type.

Ecuador. Orellana: Yasuní Research Station, on decaying wood, Mar 2013, A. Salazar s.n. (holotype: QCAM3430).

##### Description.

***Basidiocarps*** solitary or gregarious, laterally stipitate, dimidiate, spathulate to circular, woody, 15–21 × 8–11 cm; ***pileus*** surface laccate, tuberculate, glabrous, zonate reddish-brown to vinaceous-brown, upper surface covered by cinnamon-coloured powder of deposited basidiospore, margin obtuse, yellow when young changing to reddish-brown with age; ***context*** firm, yellowish-brown, duplex, with melanoid bands or deposits embedded in context tissue; ***pore surface*** white when young, blackish-brown to vinaceous-black when old, pores circular to irregular, 4–6 per mm; ***tube layers*** ochraceous-tawny to brownish-black, 10–12 mm thick. ***Stipe*** lateral, 25–35 cm long, round or slightly compressed, tuberculate or smooth, 12–18 mm diam. and with a reddish-brown, shiny cuticle. ***Hyphal system*** trimitic; contextual generative hyphae thick-walled, with clamps, hyaline, 3.5 µm in diam.; skeletal hyphae thick-walled, 1.5–6 µm in diam., light yellow; binding hyphae thin and thick-walled, 1–3.5 µm in diam. ***Cuticular cells*** club-like, yellowish, upper part with small outgrowths, with amyloid reaction with Melzer’s Reagent, 40–55 × 7–14 µm. ***Basidia*** not observed. ***Basidiospores*** ellipsoid to oblong, truncate at the distal end; with two walls, pale yellow, moderately coarsely echinulate, 8–10 × 5–7 µm. ***Chlamydospores*** not observed.

**Figure 7.**
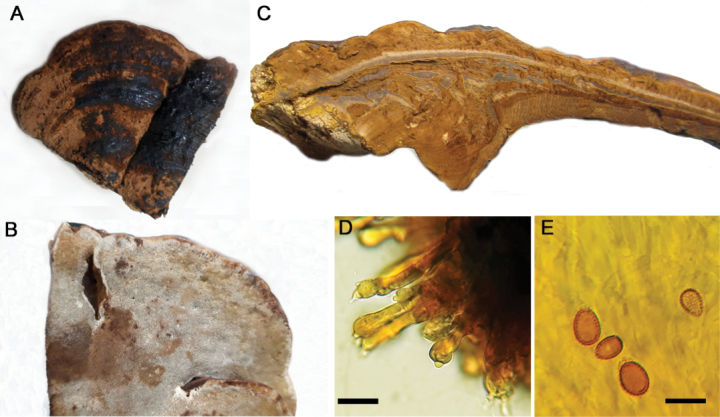
*Ganodermaecuadorense***A** basidiocarp (Mata 765) **B** pore surface (Mata 765) **C** context tissue (MMG-181) **D** cuticular cells (Mata 765) **E** basidiospores (Lopez 7241). Scale bars: 20 µm (**D**); 10 µm (**E**).

##### Descriptions and illustrations.

Crous et al. (2016).

##### Substrata.

On decaying hardwood.

##### Altitudinal distribution.

Lowlands.

##### Geographic distribution.

Brazil, Ecuador, and French Guyana. This is the first report for Costa Rica and Central America.

##### Specimens examined.

Costa Rica. Alajuela: Arenal, Parque Nacional Volcán Tenorio, sector El Pilón, 10°42'58.23"N, 84°59'15.91"W, 700 m elev., 27 Jun 1999, M. Mata Mata-765 (CR3484383). Heredia: Sarapiquí, Puerto Viejo, Estación Biológica La Selva (OET), Sendero Experimental Sur, 10°25'59.6"N, 84°0'16.2"W, 30–100 m elev., 23 Jun 2022, J. Carranza JCV 3-22/GA-52 (USJ109796, sequences ITSOQ845463); 10°25'59.5"N, 84°0'16.3"W, 100 m elev., on log, 06 Nov 2016, J. Carranza JCV 3-16 (USJ109702). Limón: Pococí, Guápiles, Zona Protectora acuíferos de Guácimo y Pococí, bosque sobre colina La Roca, 10°09'57"N, 83°47'59"W, 472 m elev., 06 Jun 2022, M. Montero MMG-181A (USJ109798, sequences ITSOQ845465); en arboleda rodeada de potreros, 10°09'55"N, 83°48'05"W, 410 m elev., 08 Sep 2022 M. Montero MMG-209 (USJ109799, sequences ITSOQ845466). Puntarenas: Cantón Central, Isla Chira, 10°6'5.01"N, 85°8'14.15"W, 0–100 m elev., 29 Jul 2005, I. López Lopez-7241 (CR3970559). Osa, Parque Nacional Corcovado, Estación Sirena, Sendero Espaveles a sendero La Olla, 8°29'12.04"N, 83°35'42.8"W, 0–30 m elev., on log, 07 Jul 2022, J. Carranza, M. Mardones, E. Fletes GA-57 (USJ109797, sequences ITSOQ845464, LSUOQ835185); Estación La Leona, 8°26'49.74"N, 83°31'10.04"W, 10 m elev., on log, 30 Aug 2014, J. Carranza JCV 2-14 (USJ109682); 8°26'49.74"N, 83°31'10.04"W, 10 m elev., on log, 16 Sep 2016, J. Carranza JCV 7-16 (USJ109691).

##### Specimens of other species examined for comparison.

*Ganodermaperzonatum*. Cuba. Santiago de las Vega, 08 Nov 1904, F.S. Earle 309 (type, NYBG 985702).

##### Discussion.

*Ganodermaecuadorense* (as *ecuadoriense*) was recently described from the Amazon Basin in Ecuador (Crous et al. 2016). It is characterised by the laterally stipitate basidiocarp, with dimidiate, laccate, reddish-brown pileus, usually covered by a cinnamon-coloured powder of deposited basidiospores. Microscopically, the main characteristics are their club-shape cuticular cells and the small (8–10 × 5–7 µm) and yellow basidiospores. We could not examine the type specimen of *G.ecuadorense*, but the morphological characteristics observed in our specimens agree well with the description in the protologue.

According to Crous et al. (2016), morphologically, *G.ecuadorense* is similar to *G.perzonatum* Murrill. The type specimen of *G.perzonatum* has a very short stipe, darker than the pileus, measuring 0.5–1 × 0.5–1.5 cm. Additionally, it has discontinuous melanoid bands; the spores are 8–10 × 6–8.5 µm and the cuticular cells do not have projections and are shorter than in *G.ecuadorense*. Steyaert (1980) considered *G.perzonatum* as a synonym of *G.parvulum*.

Sequences of four specimens from Costa Rica (GA-57, GA-52, MMG-181a, MMG-209) clustered in a subclade with *G.orbiforme* from Brazil (clade II) forming a well-supported terminal subclade (0.94/90) with sequences labelled as *G.ecuadorense* (including the type) from Brazil, Ecuador and French Guyana and *G.subfornicatum* from French Guyana. Fryssouli et al. (2020) considered *G.ecuadorense* as a synonym of *G.subfornicatum*, based on the phylogenetic analyses of the ITS region. However, *G.ecuadorense* still appears as a valid species at Index Fungorum. In the BLASTN search of our sequences, the results gave the highest score to sequences identified as *G.ecuadorense* (including the holotype).

Therefore, until more data are available, we identify our specimens as *G.ecuadorense* based on: (i) the similar morphological characteristics of our specimens with the description in the protologue of *G.ecuadorense*, (ii) the position of our ITS sequences in the phylogenetic analysis within a terminal subclade with other sequences of *G.ecuadorense* (including the holotype) and (iii) the lack of more sequences of *G.subfornicatum* (including type material) in GenBank (see Fryssouli et al. (2020) for a complete discussion on the topic).

#### ﻿6. Ganoderma oerstedii

Taxon classificationFungiPolyporalesPolyporaceae

(Fr.) Murrill, Bull. Torrey bot. Club 29: 606 (1902)

8704E0BD-A9D3-572C-BBBE-5AE1B9ECE084

Figs 3H , 8

= Ganoderma tuberculosum Murrill, N. Amer. Fl. (New York) 9(2): 123 (1908).*Type*: BELIZE (as British Honduras), 1906, M.E. Peck *s.n.* (holotype: BPI236681!).

##### Type.

Costa Rica: *s. l.*, 1846, Oersted. *s.n.* (neotype: BPI236610!).

##### Descriptions.

***Basidiocarps*** gregarious, solitary or imbricate, mostly sessile, sometimes laterally stipitate, dimidiate, ungulate or spathulate woody, rugulose, 2.8–19.1 × 2.1–24.5 × 0.7–3.9 cm; ***pileus*** surface with laccate zones, glabrous, zonate, brownish-red, vinaceous-brown, vinaceous-red, yellowish-red, gradually changing to yellowish-brown to deep yellow in the margin, margin obtuse; ***context*** firm, yellowish-brown, up to 6 cm thick, concentrically zonate, with inconspicuous horizontal bands of melanoid substances; ***pore surface*** yellowish-brown to pinkish-brown, darkening when handled, pores circular to irregular, 3–6 per mm; ***tube layers*** light brown to yellowish-brown, up to 0.9 cm thick, becoming darker with 5% KOH. ***Stipe*** glabrous, vinaceous-red or concolorous with pileus surface, with some laccate zones, 1.5–13.1 × 1.2–7.5 cm. ***Hyphal system*** dimitic or trimitic; contextual generative hyphae thick-walled, with clamps, hyaline, 5 µm in diam.; skeletal hyphae thick walled 3–9 µm in diam.; binding hyphae thin and thick-walled, 2–4 µm in diam. ***Cuticular cells*** from the pileus cylindrical, clavate, some nodulose, vesiculate and branched, thick-walled, with granulations in the apex, yellowish, with strong amyloid reaction with Melzer’s Reagent, 22–52(–100) × 6–20 µm. ***Basidia*** not observed. ***Basidiospores*** ovoid, truncate at the distal end; with two walls, connected by inter-wall pillars, subhyaline or yellowish-brown, negative in Melzer’s Reagent, (8–)11–14(–15) × (5–)8–11 µm. ***Chlamydospores*** thick-walled, reddish-brown, 23–30 × 16–21 µm.

**Figure 8.**
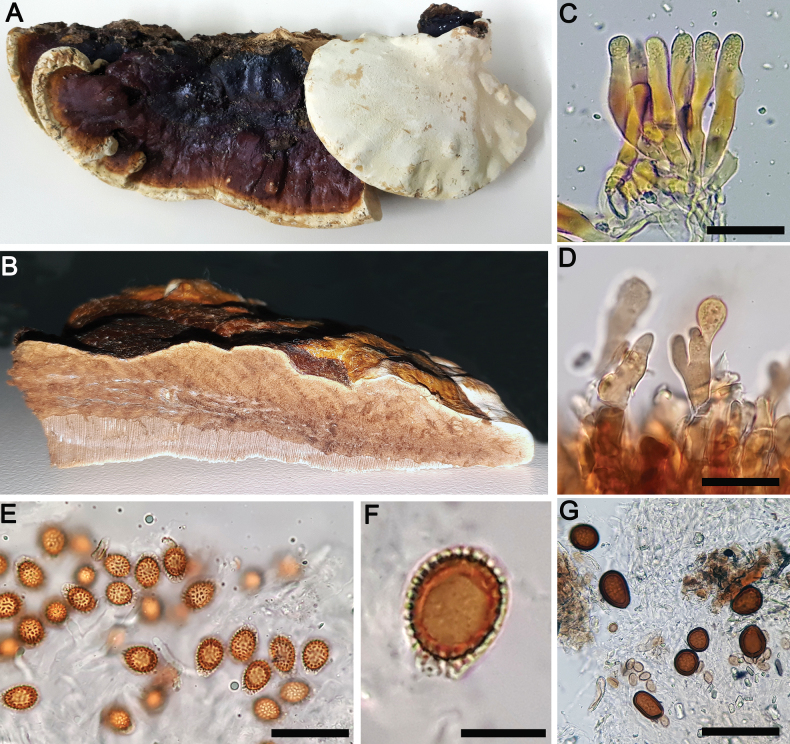
*Ganodermaoerstedii***A** basidiocarp (Cano 946) **B** context tissue (Fletes 5876) **C, D** nodulose and branched cuticular cells (Navarro 10502, Lopez 4308) **E, F** basidiospores (Fletes 5876) **G** chlamydospores (Navarro 5006). Scale bars: 20 µm (**C, D, E, G**); 10 µm (**F**).

##### Descriptions and illustrations.

Murrill (1902, 1908 as *G.tuberculosum*), Steyaert (1980), Gottlieb and Wright (1999b as *G.tuberculosum*), Ryvarden (2000, 2004), Mendoza et al. (2011), Torres-Torres et al. (2015), Lopez-Peña et al. (2016).

##### Substrata.

On living trees and logs.

##### Altitudinal distribution.

Lowlands to highlands.

##### Geographic distribution.

Widespread in the Neotropics.

##### Specimens examined.

Costa Rica. Alajuela: Grecia, Santa Gertrudis, 10°5'13.94"N, 84°17'3.96"W, 1050 m elev., 14 Jul 1991, J. Carranza JCV 16-91 (USJ33286). Guanacaste: Abangares, Higuerillas, Finca El Arboreto, 10°11'28.28"N, 85°3'10.8"W, 0–100 m elev., 20 Jun 2007, J.A.Sáenz 2049 (CR4095735); La Cruz, Parque Nacional Guanacaste, Estación Biológica Cacao, sendero Los Naranjos, 10°53'43.2"N, 85°28'24.6"W, 700–1000 m elev., 23 May 1997, E. Fletes and C. Cano 1112 (CR4130985); Santa Cruz, Reserva Ramón Alvarez, 10°17'20.4"N, 85°35'13.2"W, 0–100 m elev., 24 Sep 2011, J. Carranza JCV 7-11 (USJ83002). Heredia: Santo Domingo, San Luis, 10°0'16.4"N, 84°1'44.7"W, 1200 m elev., 06 Nov 2016, J. Carranza JCV 1-16 (USJ109683). Limón: Talamanca, Refugio de Vida Silvestre Gandoca- Manzanillo, sector Manzanillo, alrededores del Centro Operativo, 9°38'19.6"N, 82°38'56.6"W, 0–100 m elev., 26 Sep 2001, R. Valladares RValladares 555 (CR3468098). Puntarenas: Coto Brus, San Vito, Área de Conservación La Amistad Pacífico, Zona Protectora Las Tablas, Fila Chiquizá, 8°55'34.4"N, 82°46'00.95"W, 1500–1600 m elev., 19 Jul 2002, E. Navarro 5006 (CR3516656); Osa, Parque Nacional Marino Ballena, Finca Roca, a orillas de la playa, 9°9'9.02"N, 83°44'46.9"W, 0–100 m elev., 21 Jan 2004, E. Fletes 5876 (CR3813349). San José: Montes de Oca, San Pedro, Universidad de Costa Rica, Finca 1, estacionamiento del CIICLA, 9°56'19.5"N, 84°3'9.4"W, 1100 m elev., 11 Sep 2019, J. Carranza GA-21 (USJ109786); 9°56'19.5"N, 84°3'9.34"W, 1100 m elev., 18 Dec 2019, J. Carranza GA-24 (USJ109787, sequences ITSOQ845469).

##### Discussion.

This species was originally described from Costa Rica. It is characterised by its woody basidiocarp, reddish-brown in the base, to deep yellow in the margin. The species has a yellowish-brown context, with continuous resinous bands and clavate, branched and vesiculate cuticular cells with strong amyloid reaction with Melzer’s Reagent. The two walls in the basidiospores are connected by inter-wall pillars.

The piece of the neotype specimen examined under *Polyporusoerstedii* Fr. - *G.oerstedii* (Fr) Murr., collected in Costa Rica, only contained a small portion of the tubes with abundant ovoid, truncate, echinulate spores, 9.3–13.6 × 7.65–9.3 µm. Annotations done by O. Juel, Xin-Cun Wang, Donjmei Wang and Ryvarden mentioned spores 9–10 × 6.5–8 µm (with wall 11–12 µm), 11.5–13 × 8.5–10.5 µm, 11.5–15 × 8–11.5 µm (with wall), 10–13.5 × 6.5–10.5 µm (without wall) and 11–14 × 7–10 µm, respectively. The spores in the specimens studied from Costa Rica are in the range of the ones found on the neotype and the ones mentioned by the above researchers.

In taxonomic studies by Ryvarden (2000) and Torres-Torres et al. (2015) and in Mycobank (https://www.mycobank.org/), *G.oerstedii* is considered a synonym of *G.tuberculosum*, although newer studies by Loyd et al. (2018) and Fryssouli et al. (2020), as well as Index Fungorum contradicted them. We examined the type specimens of both taxa and significant morphological differences were not observed; hence, we concluded that these taxa are co-specific.

According to Loyd et al. (2018), *G.tuberculosum* generally produced sessile basidiomata. However, amongst Costa Rican specimens, we found two forms: sessile and laterally stipitate basidiomes. Additionally, Loyd et al. (2018) mentioned that chlamydospores were lacking in the species, although they are presented in our collections.

The sequences from Costa Rican specimens GA-24 and JV1607/62 (retrieved from GenBank, MZ354944) strongly supported a terminal subclade (1/99), together with other sequences labelled as *G.tuberculosum* or *G.oerstedii* collected from Brazil, Florida (USA) and Mexico, within clade I that also includes the species G. *philippii*, *G.flexipes* and *G.wiiroense*.

#### ﻿7. Ganoderma parvulum

Taxon classificationFungiPolyporalesPolyporaceae

Murrill, Bull. Torrey bot. Club 29: 605 (1902).

5F5010A9-6919-522D-96ED-102513260830

Figs 3I , 9

≡ Fomes parvulus (Murrill) Sacc. & D. Sacc, Syll. Fung. (Abellini). 17: 123 (1905). *Type*: NICARAGUA, s.d., C. L. Smith *s.n.* (type: NYBG 985699!).
= Fomes stipitatus Murrill, Bull. Torrey Bot. Club. 30(4): 229 (1903).
≡ Ganoderma stipitatum (Murrill) Murrill, N. Amer. Fl. (New York) 9(2): 122 (1908). *Type*: NICARAGUA, 1891, Smith C. L. and Shimek B.*s.n.* (isotype: NY 985679!).
= Fomes subamboinensis Henn., Hedwigia 43(3): 175 (1904) [MB148868].
≡ Ganoderma subamboinense (Henn.) Bazzalo & J.E. Wright ex Moncalvo & Ryvarden, Synopsis Fungorum 11: 82 (1997).
≡ Ganoderma subamboinense var. subamboinense Bazzalo & J.E. Wright (invalid name).

##### Description.

***Basidiocarps*** annual, stipitate or with a contracted base, woody, solitary or gregarious, applanate to sulcate, irregular to tuberculate, dimidiate to semicircular, 1.5–8 × 0.7–12.3 × 0.5–2 cm; ***pileus*** surface laccate or dull, sulcate, crustose, rugulose to glabrous, vinaceous-brown, vinaceous-black, reddish-brown, brownish-black to yellowish-brown, yellowish-red, margin obtuse, vinaceous-brown, reddish-brown, yellowish-red or yellowish-brown, azonate or with yellowish-brown, brownish-black or reddish-brown zones; ***context*** duplex, corky, yellowish-brown to beige, becoming darker, vinaceous-brown to reddish-brown, just above the tubes, with two horizontal bands of melanoid substances, sometimes more like deposits than bands, that originate from the base of the stipe, 2–17 mm thick, becoming dark with KOH; ***pore surface*** reddish-brown, vinaceous-brown to yellowish-brown, pores circular, 4–7 per mm; tube layers reddish-brown, brownish-black to yellowish-brown, sometimes whitish within; ***tubes layers*** simple to stratified, 1–8 mm thick. ***Stipe*** glabrous, sulcate or smooth, laccate or dull, lateral, vinaceous brown, vinaceous-black, vinaceous-red, yellowish-brown or brownish-black, 2.3–8.5 × 0.5–3 × 0.4–3 cm. ***Hyphal system*** dimitic; contextual generative hyphae inconspicuous, thin or thick-walled, with clamps, 4 µm; skeletal hyphae thick-walled, brown, aseptate, occasionally branched, 3–7 µm in diam. ***Cuticular cells*** from the pileus cylindrical to clavate, yellowish, with granulations and amyloid reaction on Melzer’s Reagent in the apical part, thick-walled, nodulose, 31–66 × 5–10 µm (20–40 × 6–10 µm, Ryvarden (2004)). ***Basidia*** not observed. ***Basidiospores*** ovoid, truncate at the distal end; with two walls, connected by inter-wall pillars, brown or subhyaline, negative in Melzer’s Reagent, 7–10 × 5–7 µm. ***Chlamydospores*** few, in the context, thick-walled, yellowish-brown, slightly ornamented, 6–8 × 5.5–6 µm; in pure culture, abundant, thick-walled, brown, ornamented, with longitudinal ridges, 8–10 × 6–9 µm.

**Figure 9.**
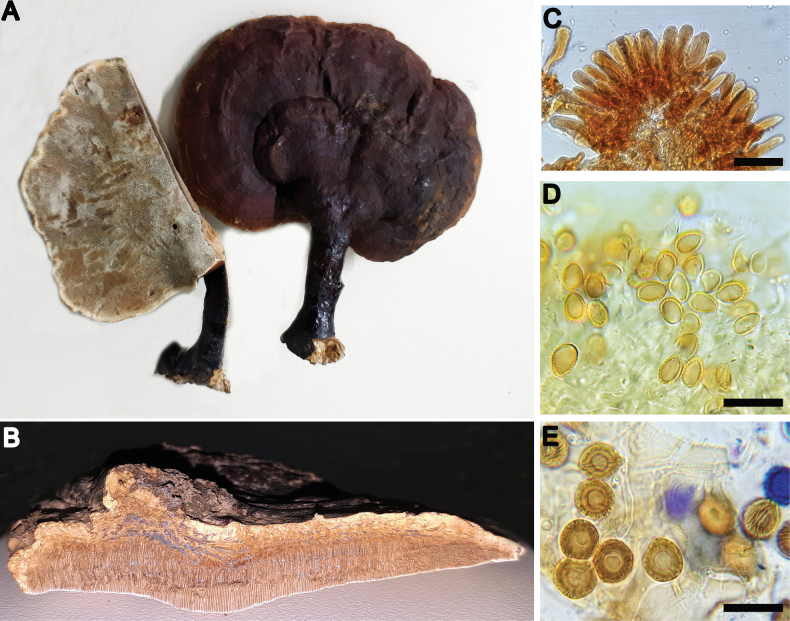
*Ganodermaparvulum***A** basidiocarp (Fletes 266) **B** context tissue (Fletes 6566) **C** cuticular cells (Fletes 266) **D** basidiospores (Fletes 6566) **E** chlamydospores (GA-09). Scale bars: 10 µm (**C**); 20 µm (**D, E**).

##### Descriptions and illustrations.

Ryvarden (2000, 2004, as *G.stipitatum*), Cabarroi-Hernández et al. (2019).

##### Substrata.

On hardwood logs.

##### Altitudinal distribution.

Lowlands to highlands. In Costa Rica, this species is more common in the lowlands.

##### Geographic distribution.

Widespread in the Neotropics, reported from south-eastern USA (Florida) to Brazil.

##### Specimens examined.

Costa Rica. Alajuela; Poás, Carrillos, 10°1'41.6"N, 84°16'55.1"W, 800 m elev., M. Mata GA-10 (USJ109860, sequences ITSOQ845473, LSUOQ835189). Cartago; Turrialba, La Amistad Caribe, Parque Nacional Barbilla, sendero El Felino, 9°58'19.7"N, 83°27'50.8"W, 700–800 m elev., 07 Aug 2002, R. Valladares 1372 (CR3537817). Guanacaste: Liberia, Parque Nacional Guanacaste, Estación Biológica Cacao, 10°55'35.4"N, 85°28'2.4"W, 1700 m elev., 4 Jul 1994, J. Carranza JCV 28-94 (USJ53210); Sector Colorado, camino a pozas del Río Colorado, 10°40'3.10"N, 85°29'12.6"W, 150 m elev., 3 Sep 2021, M. Mardones, M. Mata, J. Carranza GA-37 (USJ109790); 10°40'6.9"N, 85°29'9.01"W, 150 m elev., GA-35 (USJ109791); 10°40'5.21"N, 85°28'56.4"W, 150 m elev., GA-38 (USJ109792); GA-46 (USJ109861, sequences ITSOQ845474, LSUOQ835190). Heredia: Santo Domingo, San Luis, carretera Braulio Carrillo, 9°58'28.2"N, 84°4'4.3"W, 1200 m elev., on *Casuarina* sp., 04 Jul 2018, M. Mardones GA-04 (USJ109789, sequences ITSOQ845470, LSUOQ835187, *TEF* OR022012); 9°58'28.2"N, 84°4'4.3"W, 1200 m elev., 04 Aug 2018, M. Mardones GA-08 (USJ109714, sequence ITSOQ845471). Sarapiquí, Puerto Viejo, Estación Biológica La Selva (OET), 10°26'0.30"N, 84°0'16.8"W, 100 m elev., 23 Jun 2022, J. Carranza JCV 3-16 (USJ109702). Limón: Cantón Central, Reserva Biológica Hitoy Cerere, Sendero Tepezcuintle, 9°40'19.9"N, 83°01'42.9"W, 0–100 m elev., 9 Nov 2002, R. Valladares 1636 (CR3557538); Sixaola, 9°30'25.4"N, 82°36'43.59"W, 10 m elev., 24 Jun 1988, A. Conejo 32-88 (USJ28075). Puntarenas: Coto Brus, San Vito, Área de Conservación La Amistad Pacífico, Zona Protectora Las Tablas, Estación Biológica Las Alturas, sendero a Cerro Echandi, 8°56'56.9"N, 82°49'59.0"W, 1500–1600 m elev., 12 Nov 1999, E. Navarro 1439 (CR1546847). Golfito, Reserva de Vida Silvestre Golfito, sendero La Lechería, 8°39'17.3"N, 83°13'4.8"W, 100–200 m elev., 13 Jun 2003, E. Fletes 5248 (CR3727447); 8°39'18.1"N, 83°13'8.8"W, 100–200 m elev., 09 Feb 1991, J. Carranza JCV 4-91 (USJ33128); Sector el Tajo, 8°40'11.2"N, 83°11'55.4"W, 0–100 m elev., 05 Sep 2004, E. Fletes 6566 (CR3881862). Osa, Parque Nacional Corcovado, Rio Madrigal, quebrada Ceniza, 8°26'53.9"N, 83°30'54.6"W, 200–300 m elev., 19 Mar 2003, E. Fletes 4943 (CR3700175); Parque Nacional Corcovado, Estación Los Patos, márgenes del Rio Rincón, 8°34'27.7"N, 83°30'27.6"W, 80 m elev., 21 Aug 1999, E. Fletes 631 (CR1546789); Parque Nacional Corcovado, orillas del río Pavón, 8°31'1.03"N, 83°35'52.8"W, 100–200 m elev., 27 Feb 2005, E. Fletes 7239 (CR3932787); Parque Nacional Corcovado, Estación Sirena, márgenes del río Sirena, 8°28'51.12"N, 83°35'51.2"W, 0–100 m elev., 09 Apr 2003, E. Fletes 4999 (CR3717017); sendero Guanacaste, 8°28'56.0"N, 83°35'21.72"W, 10 m elev., 25 Mar 1999, E. Fletes 266 (CR1546586); Sendero Sirena, 8°28'47.8"N, 83°35'46.9"W, 0–30 m elev., on log, 07 Jul 2022, J. Carranza, M. Mardones, E. Fletes GA-56 (USJ109780, sequences ITSOQ845475, LSUOQ835191); Parque Nacional Corcovado, Estación La Leona, Sendero Paraíso, 8°26'49.1"N, 83°31'21.6"W, 0–30 m elev., on log, 10 Sep 2009, J. Carranza JCV 114-09 (USJ83245); Reserva Biológica Isla del Caño, sendero al mirador, 8°42'21.1"N, 83°53'27.0"W, 0–100 m elev., 20 Aug 2003, E. Navarro 7005 (CR3752717). San José: Montes de Oca, San Pedro, Campus UCR, frente a facultad de Medicina, 9°56'19.2"N, 84°3'0.2"W, 1100 m elev., on log of *Casuarina* sp., 04 Oct 1999, J. Carranza JCV 2-99 (USJ71256); 9°56'19.2"N, 84°3'0.2"W, 1100 m elev., 02 Oct 2018, M. Mardones GA-09 (USJ109788, sequences ITSOQ845472, LSUOQ835188); frente a la Facultad de Educación, on log, Nov 1999, A. Ruiz s.n (USJ71255); on log, 09 Aug 2011, J. De León, O. Morales, R. Doss JDL 15-2011 (USJ109685).

##### Specimens of other species examined for comparison.

*Ganodermapulverulentum*. Grenada. Sep 1905, W.E. Broadway s.n. (lectotype, NYBG 985708). *Ganodermasessile*. USA. New York: Westchester Co., White Plains, May 1897, L. M. Underwood s.n. (type, NYBG 985711). *Ganodermasessiliforme*. Mexico. Morelos: Cuernavaca, Gardens, and Barrancas within 3 miles of Cuernavaca, 24 Dec 1909, W. A. Murrill 392 (type, NYBG 985713).

##### Discussion.

*Ganodermaparvulum* is characterised by a laterally stipitate basidiocarp and light-coloured context on the upper part and darker close to the tubes, with melanoid encrustations or bands running from the base of the stipe (like the ones found on *G.curtisii*). According to Cabarroi-Hernández et al. (2019), ornamented chlamydospores in the context and pure culture is the only morphological characteristic distinguishing G. *parvulum* from *G.mexicanum* s.l. Few chlamydospores were observed in *G.parvulum* vouchers collected in Costa Rica and, in some specimens, were totally absent. However, in pure cultures of specimens GA-08 and GA-09, ornamented chlamydospores were numerous (Fig. 9E). In Carranza and Ruiz-Boyer (2001), chlamydospores of the culture JCV 2-99 (as *G.lucidum*) were reported as round to ovoid or elongate and 14–21 × 11–19 µm.

Cabarroi-Hernández et al. (2019) reported much larger basidiospores (11–16 × 9–14.5 µm) than those observed in the Costa Rican specimens (7–10 × 5–7 µm). The size of the basidiospores reported by Ryvarden (2000), as *G.stipitatum*, (7–9.5 × 5–6.5 µm) and Torres-Torres et al. (2012, 8–9 × 6–6.8 µm) agree with our observations. The type specimen under the name *Fomesstipitatus* Murr. collected on dead wood in Nicaragua was examined. It had very much deteriorated, with only a small portion of the pileus and context. No spores were observed, but it had cuticular cells amyloid at the apex, 19.5–24 × 6.8 µm and two melanoid bands are observed in the context. Murrill (1915) reported for *G.parvulum* spores 5 × 4 µm and for *G.stipitatum* 3.5 × 5 µm, both measurements were very small compared with those described by the above authors. The spores observed in the specimen of *G.perzonatum* considered by Steyaert (1980) as *G.parvulum* are larger, 7.7–9.4(–10) × 6–7.7(–8.5) µm, but closer to the ones found on the Costa Rican specimens and the ones reported by other researchers.

Several sequences of specimens of *G.parvulum* are represented in our dataset (GA-04, GA-08, GA-09, GA-10, GA-46, GA-56). The sequences are grouped in clade IV with good support (1/73) within a subclade containing sequences from several neotropical specimens labelled as *G.parvulum*, *G.mexicanum*, *G.stipitatum*, *G.weberianum* and *G.subamboinense*. Ganodermasubamboinensevar.subamboinense and *G.stipitatum*, neotropical species within the *Ganodermaweberianum*-*resinaceum* complex, were recently synonymised under *Ganodermaparvulum* (Cabarroi-Hernández et al. 2019).

### ﻿Excluded and doubtful species of *Ganoderma* in Costa Rica

In addition to the species previously described, there are two additional species of *Ganoderma* that may occur in Costa Rica. However, as there is not enough material or DNA sequences to confirm the identification, they are considered in this study as doubtful taxa.

#### ﻿ Ganoderma chocoense

Taxon classificationFungiPolyporalesPolyporaceae

J.A. Flores, C.W. Barnes & Ordoñez, in Crous et al., Persoonia 41: 365 (2018)

1CEC7FB4-DAE1-5F51-BFF7-F5F3F855D4AC

Fig. 3D

##### Discussion.

this species was recently described from Ecuador (Crous et al. 2018). We collected a single specimen (GA-03) in the Braulio Carrillo National Park in north-eastern Costa Rica. Macroscopical characteristics agree with the description in the protologue of *G.chocoense* (Crous et al. 2016).

The BLASTN search and the phylogenetic analyses grouped the ITS sequences of the specimen GA-03 with the sequences of the holotypes of *G.chocoense* (QCAM 3123) and *G.podocarpense* (QCAM-6422) with the highest score in similarity and strong support at the nodes (1/87), respectively. The morphological characteristics of *G.podocarpense* (Crous et al. 2017), a recently described species from Ecuador, are similar to *G.chocoense.* The distinction between both species is unclear and they are probably synonyms. Additional collections and molecular markers of both species are necessary to clarify the circumscription of these species. Considering that we only have a single specimen and the lack of basidiospores in the examined specimen, we believe it is necessary to collect more material before confirming the presence of the species in the country.

##### Specimens examined.

Costa Rica. Heredia: Santo Domingo, San Luis, Parque Nacional Braulio Carrillo, entrada San Josecito, 10°02'57.2"N, 84°01'16.6"W, 1200 m elev., 04 Jul 2018, M. Mardones, J. Carranza, M. Mata GA-03 (USJ109707, sequences ITSOQ845457, LSUOQ835181, *TEF* OR022013).

#### ﻿ Ganoderma applanatum var. laevisporum

Taxon classificationFungiPolyporalesPolyporaceae

C.J. Humphrey & Leus-Palo, Philipp. J. Sci. 45(4): 533 (1931)

97656754-D063-5D9A-9C07-B8CF68839A93

##### Discussion.

During the examination of *G.applanatum* specimens from Costa Rica, we found four relatively old specimens (JCV16-95, Navarro 8458, Navarro 3699, USJ109859) that agreed with the description of G.applanatumvar.laevisporum (Humphrey and Leus 1931; Wang et al. 2009). This species has been reported for Java, Philippines and mainland China. It is characterised by its sessile basidioma with a dull upper surface and the basidiospores with smooth wall. According to Wang et al. (2009), the species is distributed at higher elevations in the Tropics, matching with our records, since our specimens were collected above 1800 m a.s.l. The basidiospore size of our specimens (9–11 × 6–7 μm) agree with those reported by Humphrey and Leus (1931, 9.3–10.3(–10.8) × 5.4–5.9(–6.4) μm) and Wang et al. (2009, 9.2–10.5 × 5.5–6.5 μm). According to Steyaert (1972), G.applanatumvar.laevisporum is a synonym of *G.tornatum* (for a complete discussion on this topic, see Wang et al. (2009)). As we only examined four relatively old specimens, have been unable to examine the holotype and it was not possible to obtain DNA or pure cultures from them, this species is excluded from our taxonomic analysis until more specimens and molecular data are available to confirm its presence in Costa Rica.

##### Specimens examined.

Costa Rica. Alajuela: Grecia, Reserva Forestal Grecia, Bosque del Niño, sendero al acueducto, 26 June 2006, 10°8'34.62"N, 84°14'45.3"W, 1800–1900 m elev., J. Carranza JCV16-95 (USJ64962). Puntarenas: Buenos Aires, Parque Nacional La Amistad, Estación Altamira, sendero al Cerro Biolley, 9°02'21.6"N, 83°00'35.9"W, 1700–1800 m elev., 20 Jul 2004, E. Navarro 8458 (CR3866211); Estación Pittier, Sendero a Cerro Gemelo, 9°02'24.5"N, 82°57'39.9"W, 1800–1900 m elev., 18 Aug 2001, E. Navarro 3699 (CR3459327). San José: Dota, San Gerardo, Albergue de montaña Saavegre, 9°33'2.08"N, 83°48'26.31"W, 2000–2300 m elev., 09 Nov 2001, s.n. (USJ109859).

## ﻿Discussion

### ﻿Morphological and ITS-phylogenetic-species concept in *Ganoderma* species of Costa Rica

This work represents the first effort to compile the *Ganoderma* species present in Costa Rica. More than 100 specimens were examined, including previously reported taxa for the entire country. Each specimen was characterised morphologically, identified and compared with the type specimen, when available. Afterwards, the sequence data were generated to confirm the morphological identification by using phylogenetic analyses, to improve the molecular identification of the neotropical *Ganoderma* spp., based on the broadly used marker ITS (Schoch et al. 2012), in conjunction with re-description, photographs and a key for the neotropical species of *Ganoderma*.

Based on the morphological analyses, we conclude that five morphological characteristics are diagnostic within neotropical *Ganoderma* collections: (i) the distinction between stipitate and sessile basidiome; (ii) the colour of the context tissue; (iii) the presence and shape of melanised deposits in the context; (iv) the presence or absence of chlamydospores; and (v) the shape and size of the basidiospores. These findings agree with previous morphological analyses of neotropical species of *Ganoderma* (Torres-Torres and Guzmán-Dávalos 2012; Loyd et al. 2018). Some variations in the resinous deposits or melanoid bands in the context were related to the state of basidiocarp development, but it seems that they are present in all the laccate species. For example, in *G.curtisii* and *G.parvulum*, the melanoid bands are more prominent in mature specimens. Amongst non-laccate species, only in *G.australe* have these been observed. Regarding the presence/absence of chlamydospores in some species, it is important to mention that, for some species, it was necessary to confirm their presence in pure cultures because they were not always present in the basidiocarp, i.e. *G.parvulum*. In general, the chlamydospores’ characteristics have been used to distinguish species in culture and not based on the basidiocarp, where they are not always present (Adaskaveg and Gilbertson 1986).

A total of 40 consensus sequences of the ITS, LSU and *TEF* regions from Costa Rican specimens of *Ganoderma* were generated in this study. Before this study, sequences of *G.amazonense* were missing in GenBank and several other species were represented by a few sequences from North or South America. These newly-generated sequences provide data from Central American specimens that will be available for further phylogenetic studies of the genus.

On a global scale, the phylogenetic tree topology obtained in this study is mainly congruent with previously-published clade-specific phylogenies of *Ganoderma*, based on the ITS region (Moncalvo and Buchanan 2008; Loyd et al. 2018; Cabarroi-Hernández et al. 2019; Fryssouli et al. 2020; Sun et al. 2022). The ITS has demonstrated high efficacy in resolving relationships amongst terminal clades within the genus (Fryssouli et al. 2020). It has the advantage of being *Ganoderma*’s best-represented gene region in public repositories. This study resolved eight clades and 34 species or terminal clades (BPP ≥ 0.95 and BS ≥ 70). However, as several authors pointed out, the use of the ITS region is not enough to clarify the relationships at a higher level or identify complex groups (Cabarroi-Hernández et al. 2019; Sun et al. 2022). In this work, we also identified clades and species that require more molecular markers and additional taxon sampling to be resolved: (i) the phylogenetic position of *G.amazonense* and its relationship with other clades within the genus; (ii) the resolution of the terminal clade of the species *G.curtisii*; (iii) the clade comprising the neotropical species within the *Ganodermaweberianum-resinaceum* complex, specifically the circumscription of the species *G.parvulum*, *G.mexicanum*, *G.subamboinense var. subamboinense* and *G.subamboinense var. laevisporum*.

The species of *Ganoderma* previously reported for Costa Rica in studies based only on morphological data (Ruiz-Boyer 1998; Carranza and Ruiz-Boyer 2005) were consistent with the results obtained by combining morphology and ITS data. Five taxa previously reported in the country (*G.amazonense*, *G.applanatum*, *G.australe*, *G.oerstedii* and *G.parvulum*) are confirmed in this work, two more taxa are recognised in Costa Rica for the first time: *G.curtisii* and *G.ecuadorense* and the presence of the species *G.lucidum*, commonly recorded in publications on the fungi of Costa Rica, is rejected.

Before this work, there were nine ITS sequences of *Ganoderma* spp. from Costa Rica deposited in GenBank (Fig. 1). According to the position in the terminal clades of our phylogeny, they belong to *G.australe* (JMCR128), *G.parvulum* (INBFletes 7616), *G.podocarpense* (JV1504/126), *G.oerstedii* (as *G.tuberculosum*, JV1607/62) and several unidentified sequences (JMCR132, JMCR55, JMCR142, JMCR25, JMCR41), forming a terminal clade within clade VI. The only one of these species whose presence in Costa Rica was not confirmed by our morphological analyses is *G.podocarpense.* However, the validity of this species must be confirmed (see discussion of *G.chocoense*). The voucher was not deposited in an indexed collection, nor were duplicates deposited in a local collection, so examining it was not possible. On the other hand, the terminal clade that grouped the unidentified sequences correspond to ‘clade 7’ in the study of Moncalvo and Buchanan (2008) of the *G.applanatum-australe* species complex and to clade named as *Ganoderma* sp. E1 in Fryssouli et al. (2020). These sequences were grouped within a well-supported clade with the sequence of our specimen GA-27 and two sequences labelled as *G.tornatum* and *G.lobatum*. According to Fryssouli et al. (2020), the identification of the sequences as *G.tornatum* and *G.lobatum* is incorrect and the specimens should be re-examined along with the corresponding type material. Therefore, although this terminal clade could represent a new species, we assume a cautious position here until the type material is examined and more molecular markers and specimens are available.

In this study, we report seven *Ganoderma* species in Costa Rica and, with additional information obtained in further studies, the presence of at least three more species could be confirmed. Costa Rica has high species richness when compared to the number of species registered for other countries in the region with a much larger area. For example, recent studies of the genus by de Lima et al. (2014) in Brazil, Torres-Torres et al. (2015) in Mexico and Loyd et al. (2018) in the USA report 18, 12 and 13 species, respectively.

### ﻿Species of *Ganoderma* with neotropical distribution

A dichotomous key is presented for the 14 species of *Ganoderma* confirmed for the Neotropics by morphological and molecular analyses (*G.amazonense*, *G.australe*, *G.applanatum*, *G.chocoense*, *G.concinnum*, *G.curtisii*, *G.ecuadorense*, *G.martinicense*, *G.mexicanum*, *G.multiplicatum*, *G.oerstedii*, *G.orbiforme*, *G.parvulum*, *G.zonatum*).

Although we found 38 *Ganoderma* species reported in literature for the Neotropical Region, some species were not considered in the dichotomous key since: (i) lack of molecular data (*G.chalceum* (Cooke) Steyaert, *G.citriporum* Ryvarden & Iturr., *G.elegantum* Ryvarden, *G.guianense* Decock & Ryvarden, *G.longistipitatum* Ryvarden, *G.multicornum* Ryvarden, *G.nitidum* Murrill, *G.platense* Speg., *G.perzonatum*, *G.vivianimercedianum* M. Torres); (ii) recent studies confirm their distribution outside the Neotropics (*G.gibbosum*, *G.resinaceum*); (iii) doubts about the species circumscription or uncertain DNA annotation (*G.podocarpense*, *G.lobatum*, *G.tornatum*, *G.subfornicatum*); or (iv) synonymised names (*G.annulare* (Lloyd) Boedijn, *G.tuberculosum*, *G.meredithae*, *G.sessiliforme*) or transferred to other genera (*Haddowianeurospora* (J.S. Furtado) Teixeira, *Humphreyacoffeata* (Berk.) Steyaert, *Tomophaguscolossus*).

### ﻿Key to *Ganoderma* species with neotropical distribution

**Table:** 

1	Basidiocarp non-laccate, dull, stipitate, sessile or with a contracted base, yellowish-white, yellowish-brown, brownish-grey, reddish-black to brownish-black	**2**
–	Basidiocarp laccate, shiny, stipitate, sessile or with a contracted base, reddish-brown, reddish-orange or yellowish-brown	**5**
2	Basidiocarp stipitate, with contracted base or sessile, context yellowish-white, spores 8–10 × 6–7 µm	***G.amazonense***
–	Basidiocarp sessile or with contracted base, context yellowish-brown, dark brown, reddish-brown, to vinaceous-brown, spores 7–12 × 4.7–8 µm	**3**
3	Context yellowish-brown, purple-brown to vinaceous-brown, with resinous deposits or melanoid bands, spores 7–12 × 5–8 µm	***G.australe***
–	Context reddish-brown to vinaceous-brown, without resinous deposits or melanoid bands, spores 7–11 × 4.7–8 µm	**4**
4	Spores 7–10 × 5–6 µm	***G.applanatum***
–	Spores 8.9–11 × 4.7–6.4 µm	***G.chocoense***
5	Context yellowish-brown, light brown, with or without resinous deposits or with discontinuous melanoid bands	**6**
–	Context yellowish-brown, dark-brown, reddish-brown, vinaceous-brown, with resinous deposits, continuous or discontinuous melanoid bands	**10**
6	Resinous deposits or several melanoid bands present, chlamydospores absent in the context, spores 12–14 × 7–8 µm	***G.concinnum***
–	Resinous deposits or inconspicuous melanoid bands present or absent, chlamydospores present or absent in context, spores 9–15 × 5–8.4 µm	**7**
7	Chlamydospores present, melanoid bands present, spores 6.5–15 × 4.2–11 µm	**8**
–	Chlamydospores absent, melanoid bands absent, spores 11.2–15 × 5.6–8.4 µm	***G.zonatum***
8	Spores (7.5–)8–10.6 × (4.2–)6–8 µm, chlamydospores in context 8–9 × 6–7 µm	***G.mexicanum***
–	Spores 8–15 × 5–11 µm, chlamydospores in context 13.5–30 × 12.2–21 µm	**9**
9	Spores 9–13.6 × 5–8.3 µm, chlamydospores 13.5–21.1 × 12.2–17.3 µm	***G.martinicense***
–	Spores (8–)11–14(–15) × (5–)8–11 µm, chlamydospores in context, 23–30 × 16–21 µm	***G.oerstedii***
10	Context with two conspicuous melanoid bands or resinous deposits that originate from the base of the stipe, without chlamydospores, spores (9–)11–17 × (7–)8–10 µm	***G.curtisii***
–	Context with discontinuous melanoid bands or resinous deposits, spores 7–11 × 5–7 µm	**11**
11	Context yellowish-brown, vinaceous-brown, reddish-brown, with two discontinuous melanoid bands that originate from the base of the stipe, with few chlamydospores, 6–8 × 5.5–6 µm, spores 7–10 × 5–7 µm	***G.parvulum***
–	Context yellowish-brown to reddish-brown, with resinous deposits or discontinuous melanoid bands not originate from the base of the stipe, without chlamydospores, spores 7–13 × 5–8 µm	**12**
12	Context yellowish-brown to reddish-brown, cuticular cells with many irregular protuberances and outgrowths, strongly amyloid, spores 9–11.2(–13) × (–6)6.9–8.6 µm	***G.orbiforme***
–	Context yellowish-brown, cuticular cells amyloid or strongly amyloid with protuberances or apical outgrowths, spores 7–10 × 5–7 µm	**13**
13	Cuticular cells amyloid or strongly amyloid with few or many protuberances, spores 7–8.4(–10) × 5–6(–6.8) µm	***G.multiplicatum***
–	Cuticular cells amyloid with few apical protuberances, spores 8–10 × 5–7 µm	***G.ecuadorense***

### ﻿Geographic and altitudinal distribution of *Ganoderma* species in Costa Rica

Only two of the seven Costa Rican species reported here have wide ranges and pantropical distribution: *G.applanatum* and *G.australe*. *Ganodermaapplanatum* is reported by some authors as a cosmopolitan species. However, according to Ryvarden (2004), the species is not present in the Tropics, contrary to our results. Nevertheless, as mentioned above, more collections and molecular data are needed to confirm whether *G.applanatum* is present in the Neotropics or it is a closely-related species. The remaining five species seem to have geographic distribution limitations. For example, *G.amazonense* and *G.parvulum* have a restricted neotropical distribution. *Ganodermaoerstedii* is found in sub-neotropical (south Florida) and neotropical regions. *Ganodermacurtisii* has been only collected in the eastern USA and Mexico and its presence in Costa Rica is the southernmost record of this species. Similarly, *G.ecuadorense* has been reported only in tropical South America, with the report in this study being the northernmost record for the species.

On the other hand, amongst our collections, there were some different altitudinal distributions for some species (Fig. 2). For example, species such as *G.australe*, *G.oerstedii* and *G.parvulum* have been found occurring indistinctly in both lowlands and highlands. On the other hand, species such as *G.amazonense* and *G.ecuadorense* have been collected only in lowlands, between 0 to 700 m, mainly under 300 m. On the contrary, *G.curtisii* has been collected primarily in highlands above 2000 m.

## ﻿Conclusion

In conclusion, based on morphological criteria, ecological data and ITS phylogenetic analyses, we have confirmed the presence of seven species of *Ganoderma* in Costa Rica. This study clearly established the circumscription of several species which were historically combined in *G.lucidum* s.l. and broadened the distribution range of two laccate *Ganoderma* species to Central America. It also provides molecular data for three non-laccate *Ganoderma* species, i.e. *G.australe*, *G.applanatum* and G.cf.chocoense. Additionally, it lays the foundation for future studies of *Ganoderma*, focused on collecting more material and using additional molecular markers to confirm the presence of species, such as *G.chocoense* and G.applanatumvar.laevisporum in the country and to elucidate the relationships between neotropical species within the complex *G.weberianum-resinaceum*.

## Supplementary Material

XML Treatment for Ganoderma amazonense

XML Treatment for Ganoderma australe

XML Treatment for Ganoderma applanatum

XML Treatment for Ganoderma curtisii

XML Treatment for Ganoderma ecuadorense

XML Treatment for Ganoderma oerstedii

XML Treatment for Ganoderma parvulum

XML Treatment for Ganoderma chocoense

XML Treatment for Ganoderma applanatum var. laevisporum

## ﻿Additional information

### Author ORCIDs

Melissa Mardones https://orcid.org/0000-0002-4402-7817

Hector Urbina https://orcid.org/0000-0002-5570-4537

### Data availability

All of the data that support the findings of this study are available in the main text or Supplementary Information.

## Supplementary materials

Supplementary material 1 *Ganoderma* of Costa Rica_Linked Data Table Template for Primary Biodiversity Data Data type xlsx Explanation note Excel file with linked data table template for primary biodiversity data of the Costa Rican specimens examined in this work. https://binary.pensoft.net/file/935567This dataset is made available under the Open Database License (http://opendatacommons.org/licenses/odbl/1.0/). The Open Database License (ODbL) is a license agreement intended to allow users to freely share, modify, and use this Dataset while maintaining this same freedom for others, provided that the original source and author(s) are credited.Author: Melissa Mardones

Supplementary material 2 ITS alignment for global *Ganoderma* Data type fasta Explanation note ITS alignment for global *Ganoderma*, including sequences from Costa Rican specimens included in this work. https://binary.pensoft.net/file/935568This dataset is made available under the Open Database License (http://opendatacommons.org/licenses/odbl/1.0/). The Open Database License (ODbL) is a license agreement intended to allow users to freely share, modify, and use this Dataset while maintaining this same freedom for others, provided that the original source and author(s) are credited.Author: Melissa Mardones

Supplementary material 3 Bayesian Inference raw phylogenetic tree for ITS sequences of global *Ganoderma* Data type tre Explanation note Bayesian Inference raw phylogenetic tree for ITS sequences of global *Ganoderma*, performed with the program MrBayes v. 3.2.7a, and including sequences of the Costa Rican specimens generated in this work. https://binary.pensoft.net/file/935569This dataset is made available under the Open Database License (http://opendatacommons.org/licenses/odbl/1.0/). The Open Database License (ODbL) is a license agreement intended to allow users to freely share, modify, and use this Dataset while maintaining this same freedom for others, provided that the original source and author(s) are credited.Author: Melissa Mardones

Supplementary material 4 Maximum Likelihood raw phylogenetic tree for ITS sequences of global *Ganoderma* Data type result Explanation note Maximum Likelihood raw phylogenetic tree for ITS sequences of global *Ganoderma*, carried out in RAxML v.8.2.12, and including sequences of the Costa Rican specimens generated in this work. https://binary.pensoft.net/file/935570This dataset is made available under the Open Database License (http://opendatacommons.org/licenses/odbl/1.0/). The Open Database License (ODbL) is a license agreement intended to allow users to freely share, modify, and use this Dataset while maintaining this same freedom for others, provided that the original source and author(s) are credited.Author: Melissa Mardones
